# Neutrophil-derived trail is a proinflammatory subtype of neutrophil-derived extracellular vesicles

**DOI:** 10.7150/thno.51756

**Published:** 2021-01-01

**Authors:** Young-Jin Youn, Sanjeeb Shrestha, Yu-Bin Lee, Jun-Kyu Kim, Jee Hyun Lee, Keun Hur, Nanda Maya Mali, Sung-Wook Nam, Sun-Hwa Kim, Sunwoong Lee, Dong-Keun Song, Hee Kyung Jin, Jae-sung Bae, Chang-Won Hong

**Affiliations:** 1 Department of Physiology, School of Medicine, Kyungpook National University, Daegu, 41944, Republic of Korea.; 2 Department of Biochemistry and Cell Biology, School of Medicine, Kyungpook National University, Daegu, 41944, Republic of Korea.; 3 Department of Anatomy, School of Medicine, Kyungpook National University, Daegu, 41944, Republic of Korea.; 4 Department of Molecular Medicine, School of Medicine, Kyungpook National University, Daegu, 41944, Republic of Korea.; 5 Department of Pharmacology, College of Medicine, Hallym University, Chuncheon, 24252, Republic of Korea.; 6 Department of Laboratory Animal Medicine, College of Veterinary Medicine, Kyungpook National University, Daegu, 41944, Republic of Korea.; 7 Stem Cell Neuroplasticity Research Group, Kyungpook National University, Daegu, 41944, Republic of Korea.

**Keywords:** EV, extracellular vesicle, NDMV, neutrophil-derived microvesicle, NDTR, neutrophil-derived trail

## Abstract

**Aims:** Extracellular vesicles (EVs) are membrane-derived vesicles that mediate intercellular communications. Neutrophils produce different subtypes of EVs during inflammatory responses. Neutrophil-derived trails (NDTRs) are generated by neutrophils migrating toward inflammatory foci, whereas neutrophil-derived microvesicles (NDMVs) are thought to be generated by neutrophils that have arrived at the inflammatory foci. However, the physical and functional characteristics of neutrophil-derived EVs are incompletely understood. In this study, we aimed to investigate the differences between NDTRs and NDMVs.

**Methods:** The generation of neutrophil-derived EVs were visualized by live-cell fluorescence images and the physical characteristics were further analyzed using nanotracking analysis assay, scanning electron microscopic analysis, and marker expressions. Functional characteristics of neutrophil-derived EVs were analyzed using assays for bactericidal activity, monocyte chemotaxis, phenotype polarization of macrophages, and miRNA sequencing. Finally, the effects of neutrophil-derived EVs on the acute and chronic inflammation were examined *in vivo*.

**Results:** Both EVs share similar characteristics including stimulators, surface marker expression, bactericidal activity, and chemoattractive effect on monocytes via MCP-1. However, the integrin-mediated physical interaction was required for generation of NDTRs whereas NDMV generation was dependent on PI3K pathway. Interestingly, NDTRs contained proinflammatory miRNAs such as miR-1260, miR-1285, miR-4454, and miR-7975, while NDMVs contained anti-inflammatory miRNAs such as miR-126, miR-150, and miR-451a. Although both EVs were easily uptaken by monocytes, NDTRs enhanced proinflammatory macrophage polarization whereas NDMVs induced anti-inflammatory macrophage polarization. Moreover, NDTRs showed protective effects against lethality in a murine sepsis model and pathological changes in a murine chronic colitis model.

**Conclusion:** These results suggest that NDTR is a proinflammatory subtype of neutrophil-derived EVs distinguished from NDMV.

## Introduction

Extracellular vesicles (EVs) are membrane-derived vesicles surrounded by lipid bilayers 1. Since EVs express various ligands and receptors originating from their source cells and transport various molecules to their target cells, they are important mediators of intercellular communications 2. Neutrophils, the professional phagocytes, also produce EVs in response to various stimulators. Neutrophil-derived EVs are thought to be generated through the membrane blebbing and shedding, and conform to the general description of EV 2. Recently, a specialized type of neutrophil-derived EVs was discovered and called trails 3. During migration through inflamed tissue, the uropods of neutrophils are elongated by adhesion to the endothelium 4. These elongated uropods detach from the cell bodies, finally leaving chemokine-containing EVs 3. Hence, neutrophil-derived EVs can be categorized into two subtypes according to the mechanism of generation: neutrophil-derived microvesicles (NDMVs) and neutrophil-derived trails (NDTRs).

NDMVs are the classical type of neutrophil-derived EVs. Neutrophils generate NDMVs either spontaneously 5 or in response to various immunological stimuli such as bacterial stimulation 6-16, cytokines 12, 17, chemokines 13, complement components 13, 18, and antibodies 19, 20. Flow cytometric analysis and electron microscopic analysis showed that NDMVs are small enclosed vesicles less than 1 μm in diameter 13, 15, 17, 20-23 and surrounded with a double-layered membrane 5, 10. Phosphatidylserine (PS) is exposed in the outer layer of the membrane 6, 8, 13, 15, 16, 18, 20, 24-26 and various adhesion molecules, such as integrin αM 5, 6, 14, 15, 19, 23, integrin β 6, and L-selectins 5, 21, 26, are expressed on the surface. In addition, NDMVs contain protease-enriched granules 5, 6, 10, 12, 15, 19, 20, 26. Although NDMVs limit growth of bacteria by inducing bacterial aggregation 16, NDMVs exert anti-inflammatory functions in general 27. NDMVs suppress inflammatory gene expressions in natural killer (NK) cells 13, monocyte-derived dendritic cells (moDCs) 7, macrophages 8, 9, 24, 28, chondrocytes 17, and endothelial cells 11, 19. Moreover, NDMVs reduce cartilage damage in a murine model of inflammatory arthritis by enhancing TGF-β production from chondrocytes 17 and increase mortality in a murine model of sepsis by decreasing macrophage activation 29.

NDTRs are a recently identified subtype of neutrophil-derived EV. They are generated from neutrophils during migration from blood vessels into inflamed tissue 3, 4. The physical forces between neutrophils and endothelial cells elongate the uropods of migrating neutrophils 4, leading to the detachment of the tail portion 3. Since integrins mediate physical binding between neutrophils and endothelial cells, they are important for generation of NDTRs 30. The detached tail portion contains CXC-chemokine ligand 12 (CXCL12) which guides CD8^+^ T cells to the influenza-infected tissues 3.

Although extensive studies revealed the anti-inflammatory roles of NDMVs, the detailed understanding on the functions of NDTRs has not been elucidated. The most important difference between NDTRs and NDMVs is considered to be the spatiotemporal generation mechanism; NDTRs are found in tissues where neutrophils migrated, whereas NDMVs are found at the inflammatory foci where neutrophils arrived. Based on this difference, we hypothesized that NDTRs and NDMVs play different roles in modulating immune responses. Here, we show that NDTRs are proinflammatory EVs distinguished from anti-inflammatory NDMVs. Although both EVs share similar characteristics including bactericidal activity and chemoattractive effects against monocytes, NDTRs showed distinct proinflammatory miRNA profiles that induced proinflammatory macrophage polarization. Moreover, NDTRs protected mice against sepsis-induced lethality and attenuated pathological changes in mice with chronic colitis whereas NDMVs did not.

## Results

### Characterization of NDTRs

**Figure 1:**
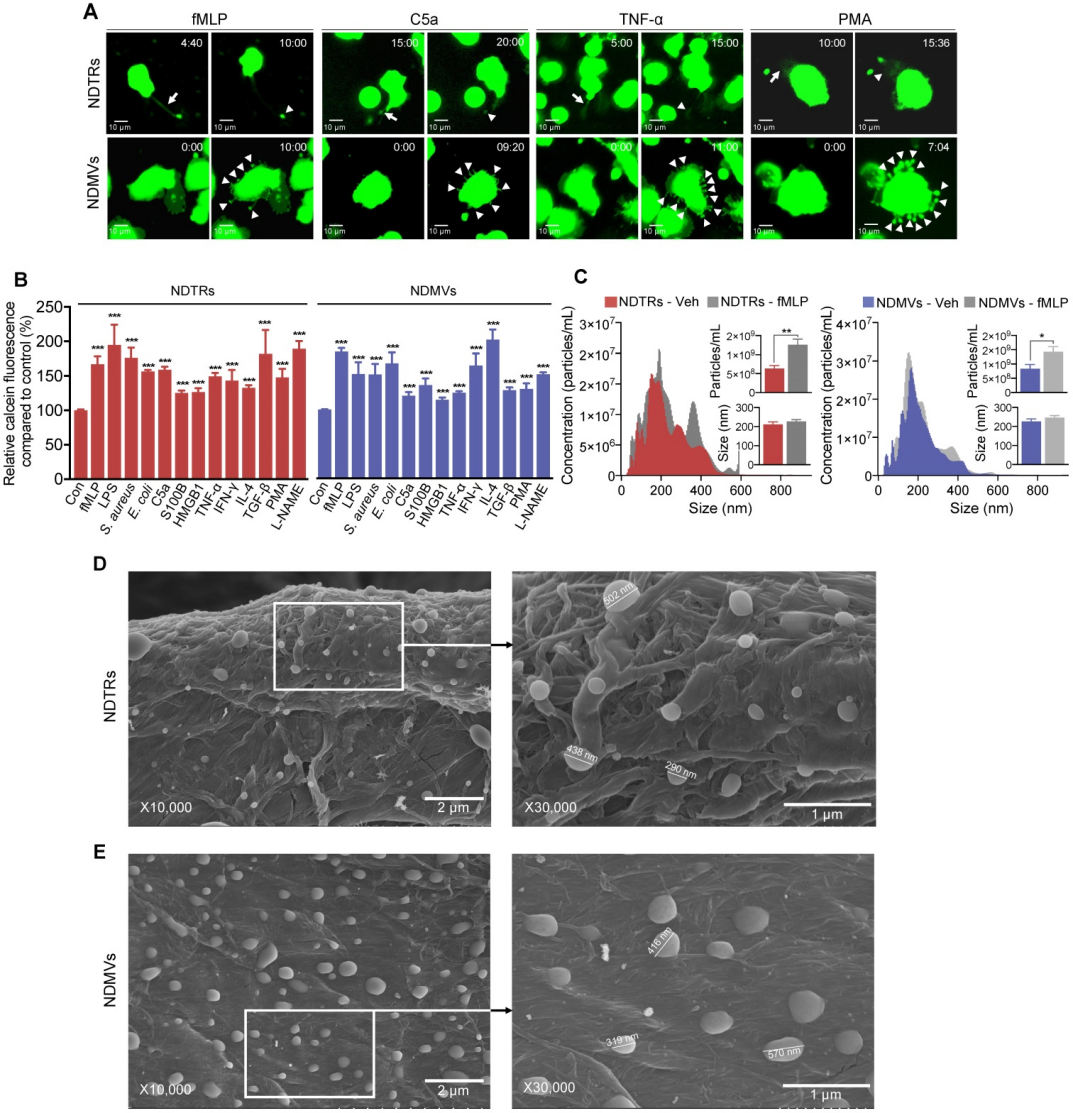
** Characterization of NDTRs.** (A-B) Various stimulators induce formation of both NDTRs and NDMVs (A) Representative time-lapse images of EVs released from neutrophils. Neutrophils were stained with cell-tracker, stimulated with the indicated stimulators, and visualized using immunofluorescence microscopy for 1 h. The time denotes minutes after stimulation. The arrows indicate elongated uropods. The arrowheads indicate deposited neutrophil-derived EVs. All data are representative of more than three independent experiments with n = 3 per each group. (B) Quantification of relative amounts of generated neutrophil-derived EVs. Neutrophils were stained with calcein-AM and stimulated with the indicated stimulators. n = 4-7 per group. Relative fluorescence normalized to the fluorescence of EVs isolated from unstimulated neutrophils. (C) Representative density plots and bar graph for sizes and concentrations of neutrophil-derived EVs. The size distribution and concentration of neutrophil-derived EVs was measured using Nanosight tracking analysis (NTA). n = 3 per group. (B-C) The data shown are the mean ± SEM. (B-C) ***P < 0.001 vs control. (D) *P < 0.05; **P < 0.01; ***P < 0.001. (D-E) Scanning electron microscopic analysis of neutrophil-derived EVs. Neutrophils were stimulated with fMLP (1 µM) and neutrophil-derived EVs were separated. Neutrophil-derived EVs were coated on filter membrane and visualized with scanning microscopy. (D) Representative picture of NDTRs. The size of particle ranged up to 502 nm. (E) Representative picture of NDMVs. The size of particle ranged up to 570 nm.

To identify specific stimulators for NDTR formation, we examined the effects of known stimulators for NDMV formation. The stimulators were categorized into the following subgroups: (i) pathogen-associated molecular patterns (PAMPs): formyl-methionyl-leucyl-phenylalanine (fMLP), lipopolysaccharide (LPS), opsonized *E. coli*, and opsonized *S. aureus*; (ii) danger-associated molecular patterns (DAMPs): high mobility group box 1 (HMGB1), complement component 5a (C5a), and S100 calcium binding protein B (S100B); (iii) inflammatory cytokines: tumor necrosis factor-α (TNF-α) and interferon-γ (IFN-γ); (iv) anti-inflammatory or immunosuppressive cytokines: tumor growth factor-β (TGF-β) and interleukin-4 (IL-4); and (v) exogenous compounds: N(γ)-nitro-L-arginine methyl ester (L-NAME) and phorbol 12-myristate 13-acetate (PMA). To visualize EV formation, neutrophils were stained with cell tracker green and live-cell fluorescence images were obtained. All stimulators induced the formation of NDTRs from neutrophils in the fibronectin-coated chemotaxis chamber (Figure 1A, Movie S1). The stimulated neutrophils exhibited elongated uropods (Figure 1A, arrows), which were subsequently detached from the cell bodies, forming NDTRs (Figure 1A, arrowheads). As expected, all stimulators induced the formation of NDMVs from neutrophils in the uncoated confocal dish (Figure 1A, Movie S2). Live-cell fluorescence imaging showed the blebbing of membranes (Figure 1A, arrowheads) and membrane blebs were finally detached, forming NDMVs (Movie S2). To quantify amounts of NDTRs and NDMVs, calcein-AM loaded neutrophils were allowed to generate EVs and fluorescence levels of calcein in neutrophil-derived EVs were measured using spectrofluorometer. A high correlation coefficient between the levels of calcein fluorescence and particle concentrations was confirmed (Figure S1A). We did not find any significant differences in amounts of neutrophil-derived EVs in response to indicated stimulators (Figure 1B).

We further characterized NDTRs according to the guidelines provided by minimal information for studies of extracellular vesicles 2018 (MISEV2018) 31. The sizes and numbers of NDTRs and NDMVs were analyzed using nanotracking analysis (NTA) assay. NTA revealed heterogeneity in the size distribution of spontaneously generated neutrophil-derived EVs (Figure 1C). The diameter of spontaneously generated neutrophil-derived EVs was between 50 and 600 nm (mode size, 73-350 nm). The sizes and diameters of neutrophil-derived EVs isolated from fMLP-stimulated neutrophils were not significantly different from those of spontaneously generated neutrophil-derived EVs [Spontaneous versus fMLP-stimulated: NDTR, 226.5 ± 14.11 nm vs 232.1 ± 16.25 nm (mean size ± SEM); NDMVs, 211.5 ± 13.47 nm vs 227.2 ± 9.324 nm (mean size ± SEM)]. The scanning electron microscopic (SEM) analysis showed the similar oval-shaped morphology of NDTRs and NDMVs (Figure 1D-E). These results suggest the robust structure of neutrophil-derived EVs, especially NDTRs, despite separation procedures.

To further investigate the mechanism underlying NDTR formation, we examined the effects of various inhibitors on the formation of NDTRs. Neutrophils were allowed to generate neutrophil-derived EVs in response to fMLP in the presence or the absence of various signaling pathway inhibitors. PD90859 (an ERK inhibitor), SB203580 (a p38 MAPK inhibitor), BAPTA-AM (a Ca^2+^ chelator), CaCCinh (a Ca^2+^-activated Cl^-^ channel transmembrane protein 16A inhibitor), Tat-C3 (a Rho inhibitor), NSC23766 (a Rac1 inhibitor), and ML141 (a Cdc42 inhibitor) attenuated the formation of both NDTRs and NDMVs (Figure S1B). However, wortmannin [a phosphatidylinositol 3-kinase (PI3K) inhibitor] significantly attenuated the formation of NDMVs but not NDTRs (Figure S1B). Moreover, IMB10 (an inhibitor of integrin αM and MAC-1) significantly inhibited the formation of NDTRs but slightly attenuated the formation of NDMVs (Figure S1B). We further confirmed these results in neutrophil-derived EVs stimulated with PMA, C5a, and TNF-α (Figure S1B). Wortmannin significantly inhibited generation of NDMVs whereas IMB10 significantly inhibited formation of NDTRs in response to stimulation with PMA, C5a, and TNF-α (Figure S1B). Since these results suggest the requirement of physical interactions for NDTR generation, we examined the effects of inhibitors on the migration of neutrophils. The accumulated distance traveled by neutrophils revealed the movement of neutrophils against known chemoattractive molecules for neutrophils such as fMLP, C5a, HMBG1, S100B, *E. coli* and *S. aureus* (Figure S1C). Interestingly, neutrophils also showed the enhanced chemokinesis against LPS, PMA, HMGB1, TNF-α, TGF-β, IFN-γ, IL-4, and L-NAME (Figure S1C). PD90859, SB205380, and IMB10 significantly inhibited movement of neutrophils against fMLP, PMA, C5a, and TNF-α (Figure S1C).

To further characterize NDTRs, we assessed the expressions of surface markers on NDTRs. Flow cytometric analysis showed NDTRs shared similar surface markers such as annexin A1 (AnxA1), phosphatidylserine [detected using annexin A5 (AnxA5)], tetraspanins (CD9, CD81, and CD63), granules (CD66b, and CD35), adhesion molecules (CD11b, CD39d, CD29, and CD18), heat shock protein (HSP70), and monocyte chemoattractant protein 1 (MCP-1) (Figure S1D). Interestingly, NDTRs showed higher expressions of CD29 (integrin β1), CD43 (leukosialin), CD49d (integrin α4), flotillin-1, and PSGL-1 (P-selectin glycoprotein ligand 1) whereas NDMVs showed higher expression of CD16 (an Fcγ type III receptor) (Figure S1D). We further investigated contents of NDTRs and found that NDTRs contained RNA and protein but not DNA (Figure S1E-F). The concentrations of proteins retained in NDTRs and NDMVs were not significantly changed according to stimulators (Figure S1G).

### NDTRs exert bactericidal activity through ROS- and granule-dependent pathway

**Figure 2:**
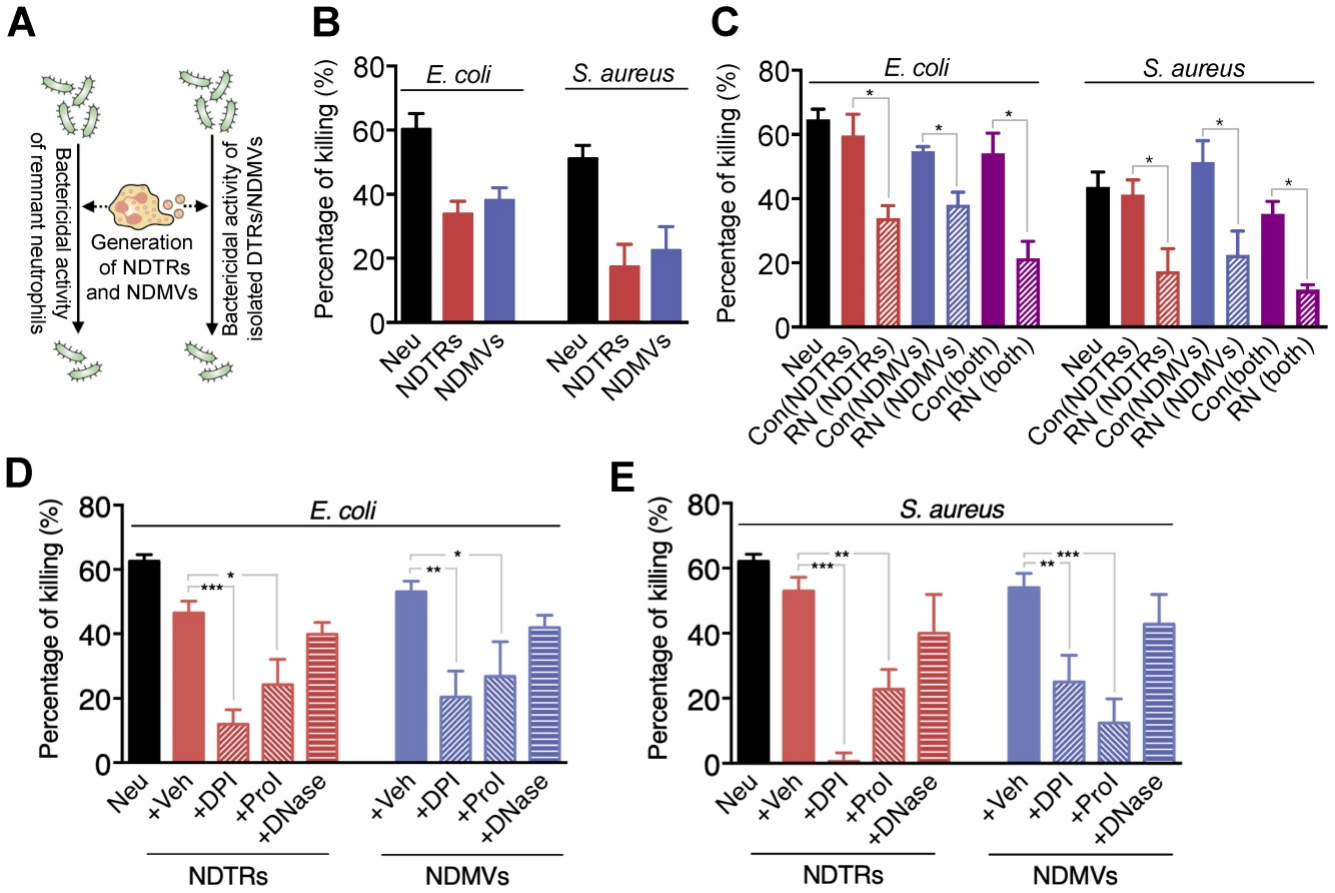
** NDTRs exert bactericidal activity via ROS- and granule-dependent pathway.** (A) Schematic depiction of experimental design. (B) Bactericidal activity of neutrophil-derived EVs. Opsonized E. coli or S. aureus were exposed to neutrophil-derived EVs separated from neutrophils stimulated with respective bacteria. (C) Bactericidal activity of remnant neutrophils (RNs) after EV formations. RNs were recovered after generation of neutrophil-derived EVs. (D-E) The effects of inhibitors on the specific pathways for bactericidal activity by neutrophil-derived EVs. Bacteria were exposed to neutrophil-derived EVs in presence of absence of indicated inhibitors. Veh, DDW; DPI, an inhibitor of NDAPH oxidase, 10 µM; ProI, protease inhibitor cocktail, 10 µM; DNase, DNase I, 10 µM. The data are pooled from three independent experiments (n = 4-7 per each group) and shown as the mean ± SEM. *P < 0.05; **P < 0.01; ***P < 0.001.

A previous study reported that NDMVs inhibit the growth of bacteria by inducing bacterial aggregation 16, hence we examined the bactericidal activity of NDTRs. *E. coli* and *S. aureus* were exposed to neutrophil-derived EVs isolated from neutrophils stimulated with equivalent amounts of *E. coli* or *S. aureus*. Then, the survival of bacteria was analyzed (Figure 2A). Both NDTRs and NDMVs showed significant bactericidal activity against *E. coli* and *S. aureus* (Figure 2B). Neutrophil-derived EVs are composed of plasma membranes and carry substantial amounts of proteins and RNA, hence, we hypothesized that the continual production of neutrophil-derived EVs might negatively influence the overall bactericidal activity of remnant neutrophils (RNs). Therefore, we examined the bactericidal activity of RNs after the generation of neutrophil-derived EVs. Neutrophils were allowed to generate either NDTRs, NDMVs, or both, and the bactericidal activity of RNs was examined (Figure 2A). Interestingly, RNs exhibited the significantly diminished bactericidal activity against *E. coli* and *S. aureus* (Figure 2C), suggesting that neutrophil-derived EVs might be involved in the general bactericidal activity of neutrophils.

Neutrophils exert their bactericidal activity through the release of reactive oxygen species (ROS), granules, and neutrophil extracellular traps (NETs) 30. We next evaluated the mechanism underlying the bactericidal activity of neutrophil-derived EVs by inhibiting each pathway of bactericidal activity. The NADPH oxidase inhibitor (diphenyleneiodonium, DPI) and protease inhibitor (PI) cocktail significantly attenuated the bactericidal activity of neutrophil-derived EVs (Figure 2D-E). DNase, an inhibitor of NETs, did not affect the bactericidal activity of neutrophil-derived EVs (Figure 2D-E). We further identified whether neutrophil-derived EVs generate ROS. A luminol assay showed that both neutrophil-derived EVs generate ROS in response to PMA stimulation (Figure S2A-B). Moreover, superoxide dismutase (SOD) activity assay and cytochrome c reduction assay showed the existence of functional units for ROS generation in neutrophil-derived EVs (Figure S2C-D). Collectively, these results suggest that both NDTRs and NDMVs exert bactericidal activity via ROS- and granule-dependent mechanisms.

### NDTRs induce monocyte chemotaxis through MCP-1

**Figure 3:**
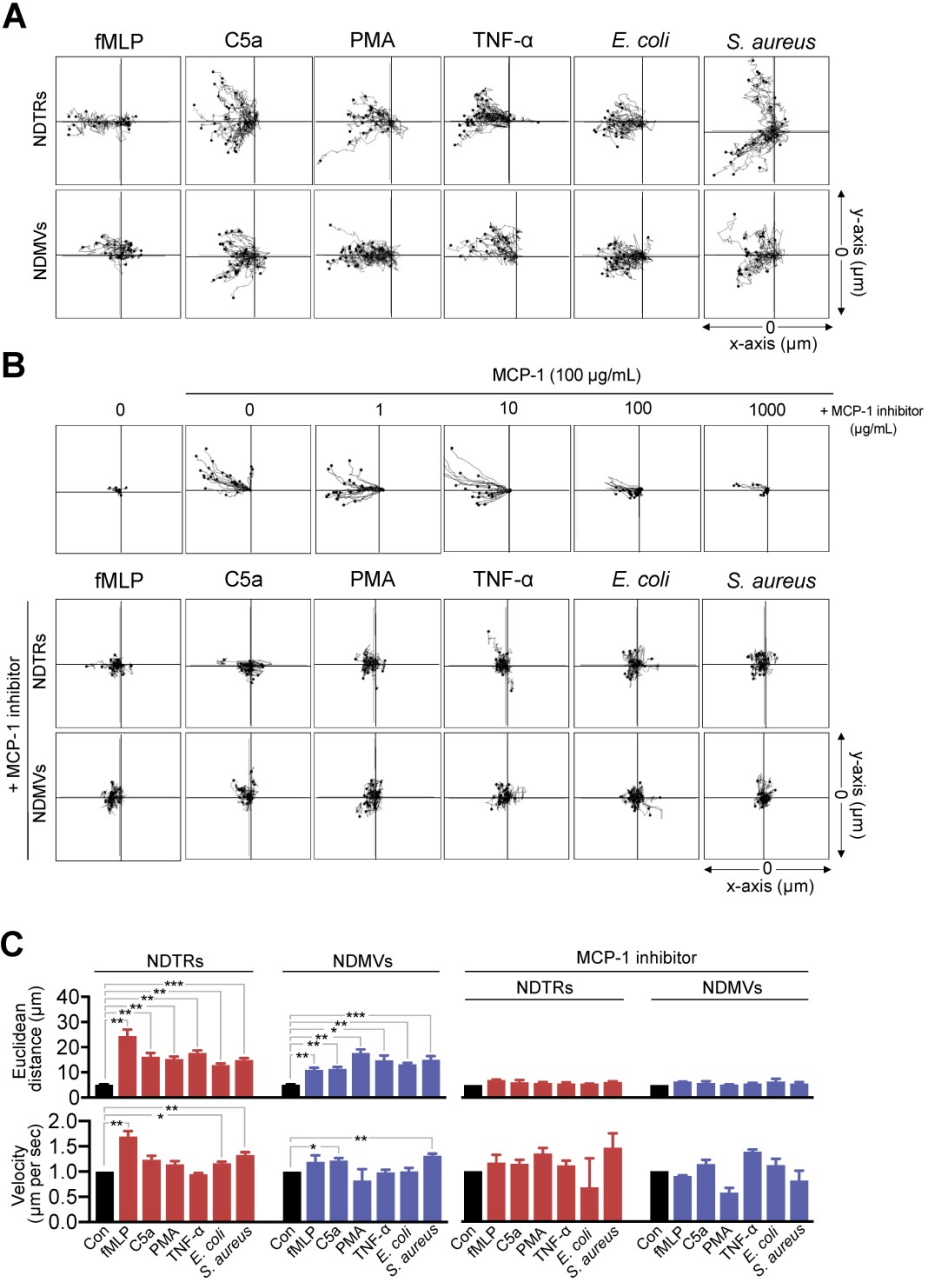
** NDTRs induce monocyte chemotaxis via an MCP-1-dependent pathway.** (A-C) Neutrophils were stimulated with indicated stimulators and EVs were isolated. The lane was coated with isolated neutrophil-derived EVs and monocytes were allowed to migrate toward isolated neutrophil-derived EVs. The distances traveled by migrating cells were tracked on every minute for 45 min. Representative tracking results of thirty cells per each group are presented. (A) Monocyte migration tracking analysis. (B) The effects of MCP-1 inhibitor on chemotaxis of monocytes against neutrophil-derived EVs. Upper panels, monocytes were allowed to migrate toward MCP-1 (100 ng/mL) in the presence or absence of CCR1 antagonist. Middle and bottom panels, monocytes were allowed to migrate toward neutrophil-derived EVs in the presence of CCR2 antagonist (+MCP-1 inhibitor, 1 µg/mL). (C) Mean distance and velocity of monocytes traveled towards neutrophil-derived EVs. The data are shown as the mean ± SEM. *P < 0.05; **P < 0.01; ***P < 0.001. All data are representative of three independent experiments (n = 3-4 per group).

Since we found the expression of MCP-1 in NDTRs and NDMVs, the effects of neutrophil-derived EVs on the chemotaxis of monocytes and macrophages were examined. One side of chemotaxis chamber was loaded with either NDTRs or NDMVs. Monocytes were isolated from peripheral mononuclear cells (PBMCs) and allowed to migrate toward neutrophil-derived EVs. Monocytes showed significant chemotaxis in a chemotaxis chamber coated with NDTRs and NDMVs (Figure 3A-C). Monocyte also showed significant chemotaxis against direct NDTR which were generated by allowing neutrophils to move toward indicated chemoattractants (Figure S3A). Macrophages were differentiated from monocytes isolated from PBMCs and allowed to migrate neutrophil-derived EVs. Macrophages did not show chemotaxis against any type of neutrophil-derived EVs (Figure S3B).

We further examined the effects of pharmacological inhibition of CCR2, a receptor for MCP-1, on monocyte chemotaxis against neutrophil-derived EVs. The CCR inhibition completely abolished the migration of monocytes toward to NDTRs and NDMVs (Figure 3B-C, +MCP-1 inhibitor). These results suggest that neutrophil-derived EVs induce monocyte chemotaxis in an MCP-1-dependent manner.

We next examined whether neutrophil-derived EVs induce chemotaxis of neutrophils themselves. Neutrophils showed efficient chemotaxis against both EVs, and LY223982 (a leukotriene B_4_ antagonist) completely abolished chemotaxis of neutrophils (Figure S3C).

### NDTRs induce proinflammatory phenotype polarization of M0-differentiated THP-1 cells

**Figure 4:**
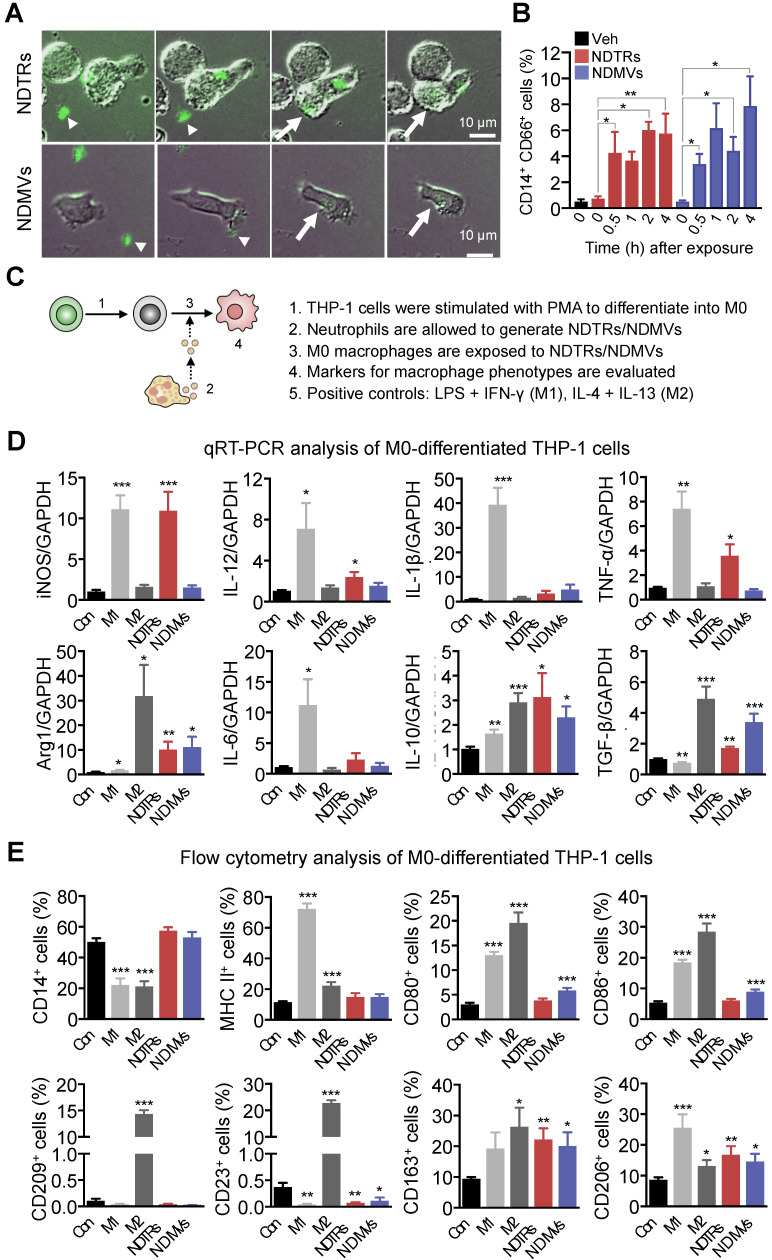
** NDTRs induce proinflammatory phenotype polarization of macrophages.** (A-B) Phagocytosis of neutrophil-derived EVs by M0-differentiated THP-1 cells. (A) Time-lapse images of M0-differentiated THP-1 cells acquiring neutrophil-derived EVs. Microfluidic chamber was coated with neutrophil-derived EVs and M0-differentiated THP-1 cells were allowed to phagocytose. Green, neutrophil-derived EVs stained with Cell Tracker Green; Arrowheads, neutrophil-derived EVs attached to the plates; Arrows, neutrophil-derived EVs phagocytosed by M0-differentiated THP-1 cells. Representative images of three independent experiments. (B) Flow cytometric analysis showing the uptake of neutrophil-derived EVs by M0-differentiated THP-1 cells. n = 3 per group. (C) Schematic depiction of experimental design for macrophage phenotype polarization. (D-E) The expressions of markers for M1 and M2 in M0-differentiated THP-1 cells exposed to neutrophil-derived EVs (n = 3-4 per each group). (D) Fold changes in the expression levels of cytokines in M0-differentiated THP-1 cells exposed to neutrophil-derived EVs using qPCR (E) The expression levels of surface markers in M0-differentiated THP-1 cells exposed to neutrophil-derived EVs using flow cytometry. The data are shown as the mean ± SEM. *P < 0.05; **P < 0.01; ***P < 0.001 compared to control.

NDMVs are considered to be generated from neutrophils in tissues 9, 10, 15, 28 whereas NDTRs are produced during migration toward inflammatory foci and guide following immune cells 3, 4. We hypothesized that NDTRs might stimulate a pro-inflammatory response by activating following immune cells while NDMVs might attenuate an excessive inflammation by suppressing neighboring immune cells. Since both EVs enhanced chemotaxis of monocytes, we compared the effects of NDTRs and NDMVs on the phenotypic polarization of monocytes into macrophages. We first examined whether monocytes directly interact with neutrophil-derived EVs. THP-1 cells, human monocytic cell line, were differentiated into M0 macrophages by stimulation with PMA (100 ngl, 48 h) and were exposed to neutrophil-derived EVs in a microfluidic chamber. M0 macrophages actively migrated toward neutrophil-derived EVs and were merged with fluorescence-stained neutrophil-derived EVs (Figure 4A, Movie S3-4). Flow cytometric analysis further confirmed the increased EV uptake of neutrophil-derived EVs in M0 macrophages (Figure 4B).

Next, we evaluated the effects of NDTRs and NDMVs on the phenotypic polarization of macrophages. M0 macrophages were exposed to either NDTRs or NDMVs and the expression of phenotypic markers were examined (Figure 4C). For comparison, M0 macrophages were incubated with either LPS/IFN-γ or IL-4/IL-13 for differentiation into M1 or M2 macrophages, respectively (Figure 4C). Interestingly, NDTRs and NDMVs differentially induced the phenotypic polarization of M0 macrophages. NDTR-exposed M0 macrophages showed increased expression of phenotypic markers of proinflammatory macrophages, such as inducible nitric oxide synthase (iNOS), IL-12, and TNF-α (Figure 4D-E). In contrast, NDMV-exposed M0 macrophages showed upregulation of phenotype markers for anti-inflammatory macrophages, such as arginase-1 (Arg-1), IL-10, TGF-β, CD163, CD206, CD80, and CD86 (Figure 4D-E). Undifferentiated THP-1 cells did not show significant changes in neither morphology nor M1/M2 markers in response to stimulations with neutrophil-derived EVs (Figure S4A-C). We further confirmed these results using primary monocytes. Peripheral blood monocytes were exposed to either NDTRs or NDMVs for 24 h and the expressions of phenotypic markers were examined (Figure S4D). NDTRs significantly enhanced the expression of iNOS and CD80 in primary monocytes whereas NDMVs significantly enhanced the arginase expression (Figure S4D). Moreover, the iNOS/Arg1 ratio was significantly enhanced in NDTR-exposed monocytes whereas it was decreased in NDMV-exposed monocytes (Figure S4D). Based on these results, we hypothesized that NDTRs and NDMVs differentially induce macrophage polarization into proinflammatory phenotype and anti-inflammatory phenotype, respectively.

To confirm the requirement of vesicular structures on effector functions of neutrophil-derived EVs, we evaluated the effects of lysed EVs. NDTRs and NDMVs were successfully lysed with SDS (Figure S4E-F) and lysed EVs showed negligible effect on macrophage polarization (Figure S4G-H). Moreover, lysed EV also did not affect bactericidal activity (Figure S4I). To examine whether diffusible molecules mediate the effects of neutrophil-derived EVs, supernatants were collected from neutrophil-derived EVs and were further filtrated. The filtrated neutrophil-derived EVs did not affect neither macrophage polarization nor bactericidal activity (Figure S4G-I). We further confirmed these results using Transwell assay. M0 macrophages loaded in upper chamber cells did not show significant changes in the expression of neither iNOS nor arginase against neutrophil-derived EVs loaded in bottom chamber (Figure S4G-H). Next, we examined whether the physical interaction is required for effector functions of neutrophil-derived EVs. Both AnxA_5_ and anti-AnxA_1_ abolished the effect of neutrophil-derived EVs on macrophages (Figure S4G-H).

### NDTRs express proinflammatory miRNAs

**Figure 5:**
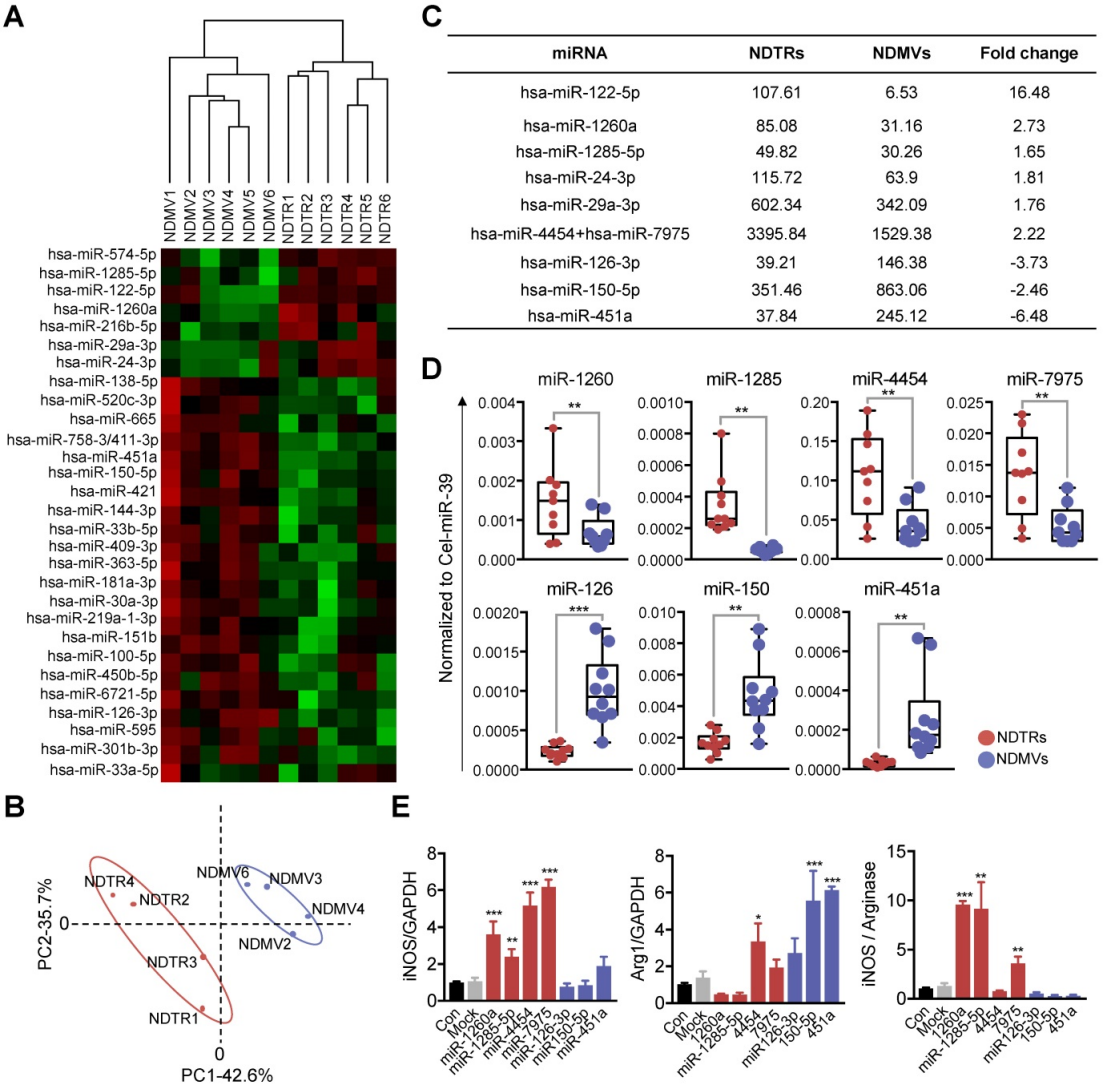
** Proinflammatory miRNA profiles in NDTRs.** (A) Hierarchical clustering of differentially expressed miRNAs in NDTRs and NDMVs. The miRNA profiles of NDTRs and NDMVs (n = 6 per group) clustered. Cluster analysis based on log10-transformed data. A red color represents relatively higher expression and a green color represents relatively lower expression. (B) Principal component analysis (PCA) plot of the miRNA expression profiles of NDTRs and NDMVs. (C) Summary of selected miRNAs differentially expressed in NDTRs and NDMVs. (D) Validation of selected miRNAs in NDTRs and NDMVs using RT-PCR (n = 10 per each group). (E) The effects of miRNA mimics (miR-1260a, miR-1285-5p, miR-4454, miR-7975, miR-126-3p, miR-150p, and miR-451a) on the expressions of iNOS and arginase in M0-differentiated THP-1 cells (n = 3 per each group). The data are shown as the mean ± SEM. *P < 0.05; **P < 0.01; ***P < 0.001.

miRNAs are small noncoding RNA molecules that post transcriptionally regulate gene expression. Various miRNAs are found in EVs and they mediate intercellular communications 32-34. Since recent studies indicate that miRNAs play a pivotal role in the phenotypic polarization of macrophages 35-37, we performed miRNA sequencing (miRNA-seq) on neutrophil-derived EVs. Agilent human miRNA microarray assay was performed on the neutrophil-derived EVs isolated from healthy volunteers (Figure 5A). The hierarchical clustering shows the differential miRNA expression patterns between NDTRs and NDMVs (Figure 5B). Furthermore, analysis of differentially expressed miRNAs (adjusted p-value < 0.05, fold change ≥ 1.5) identified 40 miRNAs with significantly different expression levels in NDTRs and NDMVs (Figure 5A). Among the top 10 differentially expressed miRNAs, six miRNAs (miR-122-5p, miR-1260a, miR-1285-5p, miR-24-3p, miR-29a-3p, and miR-4454+miR7975) were highly expressed in NDTRs, while three miRNAs (miR-126-3p, miR-150-5p, and miR-451a) were highly expressed in NDMVs (Figure 5C). Next, RT-qPCR analysis was conducted to validate the differential miRNA expression in neutrophil-derived EVs. Cel-miR-39 was added to the samples during the RNA extraction step and was used as an exogenous control for normalization. We found that four miRNAs (miR-1260, miR-1285, miR-4454, and miR-7975) were highly expressed in NDTRs, while three miRNAs (miR-126, miR-150, and miR-451a) were highly expressed in NDMVs (Figure 5D). To further confirm the involvement of miRNAs in macrophage polarization, M0-differentiated THP-1 cells were transfected with mimics of miRNAs differently expressed in NDTRs and NDMVs. Interestingly, the transfection with miRNA mimics highly expressed in NDTRs (miR-1260a, miR-1285-5p, miR-4454, and miR-7975) enhanced iNOS expressions in M0-differentiated THP-1 cells (Figure 5E). In contrast, the transfection with miRNA mimics highly expressed in NDMVs (miR-150-5p and miR-451a) enhanced arginase expressions in M0-differentiated THP-1 cells (Figure 5E).

### NDTRs exert protective effects against acute and chronic inflammation

**Figure 6:**
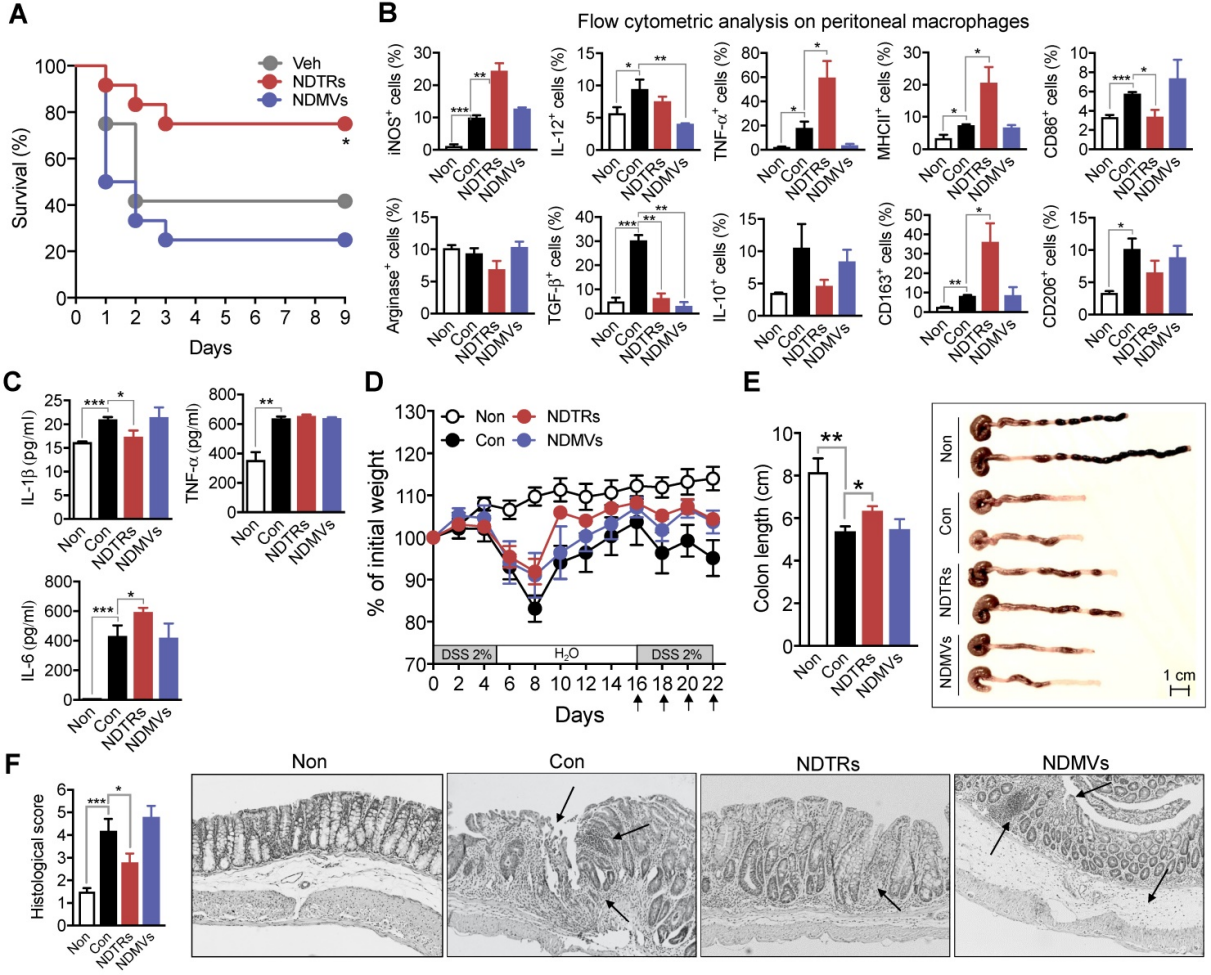
** Protective effects of NDTRs on murine models of acute and chronic inflammation.** (A) Effects of NDTRs and NDMVs in a murine model of sepsis. Experimental sepsis was induced by CLP. Survival rates of septic mice after the administration of neutrophil-derived EVs. BALB/c mice were administered an i.p. injection of either NDTRs (n = 8), NDMVs (n = 12) or vehicle (saline, n = 12) 1 h before surgery and on days 1 and 3 after surgery. *P < 0.05 compared to vehicle. (B) The expressions of markers for M1 and M2 in peritoneal macrophages isolated from septic mice injected with neutrophil-derived EVs. (C) The cytokine levels of peritoneal fluid isolated from septic mice injected with neutrophil-derived EVs. (D-F) The effects of NDTRs and NDMVs in a murine model of chronic colitis. Mice were administered with two cycles of 2% DSS for 5 days. At the beginning of the second cycle, mice were treated with NDTRs and NDMVs on every 2 days. n = 4 per each group. (D) Percentage of initial weight. (E-F) Mice were sacrificed on day 22 and subjected to evaluation. (E) Left panel, colon length. Right panel, representative photographs of the colon and cecum. (F) Macroscopic colonic damage. Left panel, histological score. Right panel, representative images of hematoxylin and eosin (H&E) staining. The arrows indicate crypt damage and inflammatory cell infiltration. The data are shown as the mean ± SEM. *P < 0.05; **P < 0.01; ***P < 0.001.

We next evaluated the effects of NDTRs and NDMVs on the murine models of acute and chronic inflammation. Cecal-ligation and puncture (CLP, a murine model of experimental sepsis) 38 and chronic dextran sulfate sodium (DSS)-induced colitis 39 were used as murine models for acute and chronic inflammation, respectively. BALB/c mice were treated intraperitoneally with either NDTRs or NDMVs 30 min prior to CLP surgery and further treated on days 1, 2, and 3 after CLP surgery. NDTR-treated mice showed increased survival, while NDMV-treated mice did not show any survival benefit (Figure 6A). Peritoneal macrophages isolated from NDTR-injected mice showed higher expressions of iNOS, TNF-α, MHC II, and CD163 with decreased expression of CD86 and TGF-β compared to peritoneal macrophages isolated from control mice (Figure 6B). Peritoneal macrophages isolated from NDMV-injected mice showed decreased expression of IL-12 and TGF-β (Figure 6B). We further examined cytokine levels in peritoneal fluid. NDTR injection attenuated the levels of IL-1β and enhanced the levels of IL-6 (Figure 6C). NDMV injection did not affect the peritoneal cytokine levels (Figure 6C). For establishment of a chronic DSS-induced colitis, mice were orally administered with two cycles of 2% DSS as previously described 39. Either NDTRs or NDMVs were administered intraperitoneally on days 16, 18, 20, and 22 (Figure 6D). Interestingly, NDTRs significantly attenuated DSS-induced reduction of colon length, while NDMVs had no effect (Figure 6E). Moreover, NDTR treatment significantly reduced colon damage (Figure 6F).

Previously, neutrophil-derived EVs were suggested as markers and mediators in sepsis 40. Therefore, we examined the existence of NDTRs and NDMVs in serum of sepsis patients to confirm the clinical relevance of neutrophil-derived EVs. Serum was obtained from 12 septic patients admitted to the intensive care units due to community-acquired pneumonia. EVs were isolated using ExoQuick and further stained with AnxA_5_ (FITC), anti-CD16 antibody, and anti-CD66 antibody. Interestingly, the percentages of both NDTRs (CD66b^+^ AnnA_5_^+^ CD16^-^) and NDMVs (CD66b^+^ AnnA_5_^+^ CD16^+^) were higher in serum of sepsis patients than in those of healthy volunteers (Figure S6).

## Discussion

In this study, we investigated the characterization and function of NDTRs. NDTRs share many similarities with NDMVs. NDTRs were generated by stimulators for NDMVs (Figure 1A-B) and showed similar physical characteristics such as diameter (Figure 1C), morphology (Figure 1D-E), and surface marker expressions (Figure S1D). Both NDTRs and NDMVs exerted bactericidal activities (Figure 2), induced monocyte chemotaxis through MCP-1 (Figure 3) and were uptaken by M0-differentiated THP-1 cells (Figure 4A-B). However, the generation mechanism of NDTRs preferentially depended on integrin-mediated signaling whereas that of NDMVs depended on PI3K signaling (Figure S1B). Phenotypically, NDTRs generally showed higher expression levels of PSGL-1 whereas NDMVs showed higher expressions of CD16 (Figure S1D). Notably, NDTRs showed higher expressions of proinflammatory miRNAs (Figure 5) and induced proinflammatory phenotype polarization of macrophages (Figure 4D-E). Finally, NDTRs showed beneficial effects in murine models of acute and chronic inflammation whereas NDMVs did not (Figure 6).

Although we searched for specific stimulators for NDTRs, most immunological stimuli induced both NDTR and NDMV formations, suggesting that the generation of NDTRs and NDMVs is not determined by stimulators that neutrophils encounter during the inflammatory process. However, we found a specific mechanism underlying the generation of neutrophil-derived EVs. Although neutrophil-derived EVs shared common mechanisms of generation (e.g., via ERK MAPK, p38 MAPK, Rho, Rac1, Cdc42, and extracellular Ca^2+^), the integrin signaling and the PI3K pathway were required for the generation of NDTRs and NDMVs, respectively (Figure S1B). These results suggest that the generation of NDTRs and NDMVs by neutrophils is dependent on the immunological environment such as physical interaction through adhesion molecules, not the type of stimulation. We next tried to identify the specific markers of NDTRs. Although NDTRs shared most markers with NDMVs, we found surface markers differently expressed in neutrophil-derived EVs. NDMVs expressed relatively higher levels of CD16 whereas NDTRs expressed relatively higher levels of PSGL-1. CD16, an Fcγ type III receptor, is found on the surface of neutrophils 41 and is preferentially distributed on the leading edge in migrating neutrophils 42. In contrast, PSGL-1 is found in the surface of resting neutrophils and redistribute to the uropods in response to chemoattractant stimulation 43. NDTRs are generated from elongated uropods of migrating neutrophils whereas NDMVs are generated from membrane blebbing from neutrophils. Hence, the contribution of this physical characteristic to the generation mechanism might be responsible for differential expression patterns of these markers.

Both NDTRs and NDMVs killed bacteria via ROS- and granule-dependent mechanism (Figure 2). Although a previous study showed that NDMVs limit bacterial growth by inducing bacterial aggregation 16, the specific mechanism underlying the bactericidal activity of NDMVs is incompletely understood. Both NDTRs and NDMVs showed bactericidal activity via ROS- and granule-dependent mechanisms (Figure 2). Previous studies have reported that NDMVs can actively generate ROS 19, 26 and contain neutrophil granules such as myeloperoxidase 5, 15, 19, lactoferrin, elastase, and proteinase 5, 6, 10, 12, 19. Consistent with this hypothesis, we found that NDMVs are equipped with functional units of ROS generation and they actively generate in response to stimulation (Figure S2). Moreover, the inhibition of NADPH oxidase and proteases significantly attenuated the bactericidal activity of NDMVs (Figure 2D-E). Interestingly, NDTRs also showed bactericidal activity via ROS- and granule-dependent mechanisms (Figure 2D). Additionally, NDTRs actively generated ROS in response to PMA stimulation (Figure S2) and expressed various granule markers (Figure S1C). Moreover, the bactericidal activity of NDTRs was also significantly attenuated by the NADPH oxidase inhibitor and protease inhibitors (Figure 2D-E), suggesting that NDTRs not only guide the migration of immune cells to inflammatory foci but also provide a defense against pathogens. Moreover, our results suggest that neutrophil-derived EVs might be involved in the general bactericidal activity of neutrophils (Figure 2C). After generation of EVs, neutrophils exhibited a marked decrease in overall bactericidal activity, suggesting that the generation of EVs leads to exhaustion state of neutrophils (Figure 2C). Neutrophils are exposed to various bacteria-derived products which are strong stimulators of neutrophil-derived EVs production. Therefore, our results suggest the possible involvement of neutrophil-derived EVs in bactericidal process of neutrophils.

Previous studies reported that NDMVs induce anti-inflammatory functions in neighboring cells. NDMV-exposed NK cells exhibited increased expression of anti-inflammatory cytokines and NDMVs inhibited the maturation of monocytes into dendritic cells 13. Moreover, NDMVs inhibit proinflammatory cytokine expression in monocytes, macrophages 8, 9, 23, 28, and endothelial cells 11, 12. In contrast, very little is known regarding the effects of NDTRs in cell-to-cell communications except that they guide following CD8^+^ T cells and monocytes toward inflammatory foci 3. We found that neutrophil-derived EVs play a pivotal role in recruiting monocytes and skewing the differentiation of M0-differentiated THP-1 cells into either proinflammatory or anti-inflammatory phenotype. Both subtypes of neutrophil-derived EVs induced monocyte chemotaxis (Figure 3) and were uptaken by M0-differentiated THP-1 cells (Figure 4A, B). Neutrophil-derived EVs showed differential effects on the phenotypic polarization of macrophages; NDTRs induced polarization of M0 macrophages toward a proinflammatory phenotype, whereas NDMVs induced of M0 macrophages polarization toward an anti-inflammatory phenotype (Figure 4C-E).

We found that miRNAs differentially expressed in NDTRs and NDMVs are responsible for macrophage polarizations into different phenotypes (Figure 5). Interestingly, most miRNAs found in NDTRs are associated with proinflammatory responses of macrophages, whereas most miRNAs found in NDMVs are associated with anti-inflammatory responses of macrophages. miR-1260a and miR-1285-5p are expressed in bacteria-infected macrophages 44-46. miR-122-5p and miR-29a-3p induce proinflammatory gene expression in macrophages 47, 48. Moreover, miR-24-3p and miR-4454 are found in monocyte-derived dendritic cells 49 and alveolar macrophages 50. In contrast, miR-126-3p and miR-150-5p are expressed in M2 polarized macrophages 51, 52 and are associated with suppression of inflammation 53-56. We identified differential miRNA expressions in NDTRs and NDMVs, suggesting that neutrophils might incorporate different types of miRNAs into EVs according to the immune environments. Since the adhesion molecules differentiate the generation mechanism of NDTRs and NDMVs, signaling associated with adhesion molecules could be helpful to understand this phenomenon.

Although neutrophils are considered to generate different types of EVs according to the immune environment 6, 16, 22, 57, the reason has not been fully understood. Successful inflammation requires effective initiation and resolution. Since neutrophils are the first cells recruited to sites of inflammation, they play a pivotal role in initiating inflammation 30, 58, 59. Neutrophils actively participate in eliminating pathogens, guide following immune cells by releasing various kinds of chemokines and cytokines, and modulate the functions of neighboring immune cells 27, 59. Neutrophils also play an important role in the resolution of inflammation; they can persist at inflammatory foci during the entire process of inflammation and participate in the resolution of inflammation by releasing proresolving factors 60. The most prominent difference between NDTRs and NDMVs is the spatiotemporal generation mechanism; NDTRs are generated from neutrophils migrating toward inflammatory foci, whereas NDMVs are thought to be generated from neutrophils that have arrived at inflammatory foci. Therefore, NDTRs might augment the proinflammatory responses of accompanying immune cells to combat imminent inflammatory insults, whereas NDMVs might limit excessive inflammation by enhancing the anti-inflammatory responses of immune cells at inflammatory foci. Interestingly, NDTRs and NDMVs showed differential effects in murine models of inflammation. NDTRs reduced lethality in the murine model of sepsis (Figure 6A) and pathological changes in the murine model of chronic colitis (Figure 6D-F). Although bactericidal activity of neutrophil-derived EVs might explain the beneficial effects of NDTRs in murine models of inflammation, we did not find any beneficial effect of NDMVs despite their bactericidal activity. A possible explanation of this contradictory effects is the differential effects of neutrophil-derived EVs on macrophages. Immune responses in sepsis composed both proinflammatory and anti-inflammatory responses. Excessive immune responses such as cytokine storm are detrimental to patients with sepsis and either dysregulated or compromised immune responses are also detrimental to hosts 61. Macrophages play a pivotal role throughout pathogenesis of sepsis. Although macrophages are responsible for excessive production of proinflammatory cytokines during sepsis, they also show immune paralyzed phenotype during sepsis 61. Indeed, the induction of proinflammatory macrophages protect against CLP-induced lethality 62, 63. NDTRs contained proinflammatory miRs and induced proinflammatory phenotype polarization of macrophages, hence this might explain beneficial effects in murine model of inflammations. In contrast, NDMVs showed negligible effects on the overall outcomes in our *in vivo* inflammation models despite their effects on macrophages. However, previous study showed the beneficial anti-inflammatory effect of NDMVs in chronic inflammation. The intra-articular injection of NDMVs decreased cartilage damage in a murine model of inflammatory arthritis by enhancing TGF-β production from chondrocytes 17. Since our study showed that NDMVs promote anti-inflammatory macrophage polarization, it could be additional mechanism underlying the beneficial effect of NDMVs on inflammatory arthritis. However, additional *in vivo* studies are needed to verify the anti-inflammatory effects of NDMVs. Moreover, neutrophil-derived EVs, exosome, exacerbate chronic inflammatory disease such as chronic obstructive pulmonary disease and bronchopulmonary dysplasia 64, hence studies regarding therapeutic application of neutrophil-derived EVs should be addressed carefully.

In conclusion, our study provides important insight into the differential functions of neutrophil-derived EVs; proinflammatory NDTRs and anti-inflammatory NDMVs. NDTRs guide monocytes and induce polarization toward proinflammatory macrophages, thereby contributing to the effective initiation of inflammation. On the other hand, NDMVs have similar characteristics to NDTRs, but they induce macrophage polarization into an anti-inflammatory phenotype. Therefore, modulating neutrophil-derived EV generation might be a new strategy for control of inflammation.

## Material and methods

### Neutrophil isolation

Human blood experiment was approved by Institutional Research Board of Kyungpook National University and Hallym University. Neutrophils were purified using histopaque (Sigma-Aldrich) centrifugation followed by Dextran sedimentation as described previously 65. Briefly, venous blood was drawn from healthy male volunteers, layered over histopaque 1077 (Sigma-Aldrich), and centrifuged at 1400 × g for 30 mins at RT. Neutrophil-containing layer was collected and sedimented with 5% (w/v) dextran (Pharmacosmos) for 45 min at 4 °C. Neutrophil-rich layer was collected and remaining red blood cells were removed using hypotonic lysis. Neutrophils were finally resuspended and incubated in RPMI 1640 (Gibco) supplemented with 5% Fetal bovine serum (FBS, HyClone) for maintaining neutrophil function. The purity of neutrophils was consistently greater than 95% determined by Wright-Geimsa staining. All the experiments regarding NDTRs and NDMVs were conducted in RPMI-1640 supplemented with 5% FBS unless otherwise indicated.

### Live cell imaging of NDMV and NDTR formation

Neutrophils (5 × 10^6^ cells) were stained cell tracker green (1.5-2.0 μg/mL, Invitrogen) and incubated in either μ-slide chamber (Ibidi) pre-coated with fibronectin (5 μg/mL, Merck Millipore) for evaluation of NDTR formation or in confocal plates for evaluation of NDMV formation. Neutrophils were stimulated with various stimulators: fMLP (1 μM, Sigma-Aldrich), LPS (1 μg/mL, Sigma-Aldrich), C5a (50 ng/mL, Sino Biologicals), S100B (100 ng/mL, Sino Biologicals), HMGB1 (100 ng/mL, Sino Biologicals), TNF-α (50 ng/mL, Sino Biologicals), IFN-γ (100 ng/mL, Sino Biologicals), TGF-β (20 ng/mL, Sino Biologicals), IL-4 (20 ng/mL, Sino Biologicals), PMA (100 μg/mL, Sigma-Aldrich), and N omega-Nitro-L-arginine methyl ester hydrochloride (L-NAME, 10 μM, Tocris bioscience). Then, EV formations were visualized by immunofluorescence microscopy (Olympus IX83, Olympus) at 37 °C, 5% CO_2_ for 1 hour.

### Separation of neutrophil-derived EVs

For separation of NDTRs, neutrophils (5 × 10^6^ cells) were seeded on culture plates coated with fibronectin (5 μg/mL, Merck Millipore) and stimulated with stimulators for 20 min. Neutrophils and media were discarded from fibronectin-coated plated and adherent NDTRs were recovered using cell scraper. For separation of NDMVs, neutrophils (5 × 10^6^ cells) were seeded on the culture plate and stimulated with stimulators for 20 min. Supernatants were collected from uncoated plates and were subjected to purification. The remnant neutrophils and debris were further removed by centrifugation at 700 × g and filtration through 1.2 μm filter (Minisart Syringe Filter, Sartorius). The filtered EV-containing supernatants were concentrated using ultra-centrifuged at 100,000 × g for 60 min at 4 °C. The concentrated EVs were dissolved in 100 μL RPMI and stored at -70 °C (maximum for 15 days). According to the concentration of neutrophils used in each experiment, EVs isolated from equivalent concentrations of neutrophils were used. The apoptotic rates of neutrophils at the time of EV harvest was examined using annexin V/propidium iodide (PI) apoptosis assay. Flow cytometric analysis showed that more than 95% of neutrophils were negative for annexin V/PI staining, which were general apoptotic rates of neutrophils incubated less than five hours. Since our experimental protocol provide neutrophil-derived EVs at high recovery with low specificity, vehicles were prepared for neutrophil non-containing groups. Vehicles for NDMVs were prepared from supernatants of media without neutrophils. Vehicles for NDTRs were prepared from scrapping fibronectin-coated plates without neutrophils. Then vehicles were centrifuged, filtered through 1.2 μm filter, and concentrated using ultra-centrifugation. No visible pellets were found in both vehicles, and they were suspended in 100 μL phenol red-free RPMI and employed as vehicles.

### Quantification of NDMV and NDTR

Neutrophils (5 × 10^6^ cells) were stained with calcein-AM (20 μg/mL, Merck Millipore) and stimulated with various stimulators: fMLP (1 μM), LPS (1 μg/mL), *E. coli* (1 × 10^6^ cells), *S. aureus* (1× 10^6^ cells), C5a (50 ng/mL), S100B (100 ng/mL), HMGB1 (100 ng/mL), TNF-α (50 ng/mL), IFN-γ (100 ng/mL), TGF-β (20 ng/mL), IL-4 (20 ng/mL), PMA (100 μg/mL), and L-NAME (10 μM). Then neutrophil-derived EVs were isolated and the fluorescence were measured using Spectramax M2/e fluorescence microplate reader (Molecular Devices).

### Characterization of NDTRs using scanning electron microscopy and nanoparticle tracking analysis

EVs generated from neutrophils (2 × 10^9^ cells) were examined using scanning electron microscopy (SEM) as previously described 66. Briefly, EVs were absorbed on the surface of filter disc, incubated in 3% glutaraldehyde (Sigma-Aldrich), dehydrated using ethanol (Merk Millipore), dried with liquid CO_2_, and sputtered with osmium. Samples were visualized using Hitachi SU8229 scanning electron microscope (Hitachi). Neutrophil-derived EVs were characterized using Nanoparticle tracking analysis (NTA). EVs were re-suspended in PBS and analyzed with the Nanosight LM10 nanoparticle characterization system (Malvern Instruments). Samples were manually injected into the sample chamber and each sample was measured in duplicate with an acquisition time of 30 sec and detection threshold setting 7. At least 975 frames were analyzed per video. NTA analytical software version 3 was used for capturing and analyzing data.

### Bactericidal activity

Neutrophils (10^8^ cells) were stimulated with either opsonized *E. coli* (DH5α, 10^7^ cells, American Type Culture Collection, ATCC) or *S. aureus* (10^7^ cells, ATCC) for 30 min on the either uncoated culture plate or fibronectin-coated culture plate. EVs were isolated according to the procedure as described except the exposure to penicillin-streptomycin (Pen-Strep, Sigma-Aldrich) for 30 min before ultracentrifugation. Either *E. coli* (10^9^ cells) *and S. aureus* (10^9^ cells) were opsonized for 30 min with autologous serum (100 μL). Opsonized E. coli (10^7^ cells) and S. aureus (10^7^ cells) were exposed to either NDTRs and NDMVs (2 × 10^9^ particles per each group) which were isolated from neutrophils stimulated with respective bacteria for 30 min. Bacteria were washed and seeded on the agar plate. For comparison of bactericidal activity, bacteria were exposed to neutrophils (10^8^ cells) for 30 min, cells were lysed with chilled DDW, and remnant bacteria were seeded on agar plates. The percentages of killing were calculated as: percentages of killing (%) = [1 - (colony counts in experimental group/colony counts in vehicle (DDW)-treated group) × 100]. For inhibition of specific bactericidal pathways, bacteria were exposed to neutrophil EVs in presence of either protease inhibitor cocktail (10 μM, Sigma-Aldrich), diphenylene iodonium (DPI, 10 μM, Molecular Probes), or DNase I (10 μM, Bio basic). For bactericidal activities of remnant neutrophils, neutrophils (10^8^ cells) were allowed to generate either NDTRs or NDMVs in presence or absence of stimulation with respective bacteria. Then neutrophils were carefully recovered by removing of EVs using centrifugation at 1400 × g, treated with pen-strep for 30 min, and the bactericidal activities of recovered neutrophils (10^8^ cells) against bacteria (10^7^ cells) were examined. For generation of remnant neutrophils after generation of both EVs, neutrophils were first allowed to generate NDTRs against bacteria, neutrophils were recovered, and were further allowed to generate NDMVs against respective bacteria. Remnant neutrophils were recovered and subjected to bactericidal assay. For generation of control neutrophils after NDTR formation [Con (NDTR)], neutrophils were allowed to adhere on the fibrinogen-coated plates, treated with vehicles (either DDW or DMSO), and harvested with gentle scrapping. For generation of control neutrophils after NDMV formation [Con (NDMV)], neutrophils were suspended in media without stimulation and harvested. For generation of control neutrophils after NDTR and NDMV formation [Con (both)], neutrophils were harvested from fibrinogen-coated plates, resuspended in media without stimulation, and harvested.

### Monocyte isolation

Monocytes were obtained from peripheral blood mononuclear cells (PBMCs) layer obtained after histopaque centrifugation by using percoll solution as described previously 67. PBMCs were layered on hyper-osmotic percoll solution (GE healthcare Life science) and further purified using iso-osmotic percoll solution. Macrophages were differentiated from monocytes by incubation with granulocytes-macrophage colony stimulating factor (GM-CSF, 10 ng/mL, Bio Legend) in RPMI supplemented with 5% FBS for 5 days. Cells (10^6^ cells) were exposed to either NDTR (10^8^ particles) and NDMV (10^8^ particles) for 24 h. For comparison, cells were exposed to either LPS (100 ng/mL) and IFN-γ (10 ng/mL) for M1 polarization or IL-4 (10 ng/mL) and recombinant human IL-12 (10 ng/mL, Sinobiologicals) for M2 polarization.

### Chemotaxis assay

Migration of monocytes against neutrophil-derived EVs were determined using μ-slide chamber (ibidi) following manufacturer's instruction. One side of chemotaxis chamber was loaded with either NDTRs or NDMVs, and monocytes were allowed to migrate. For comparison, chemotaxis chamber was loaded with MCP-1 (100 μg/mL), and migration of monocytes were measured. For inhibition of monocyte chemotaxis against EVs, cells were pre-treated with CCR2 (a receptor for MCP-1) inhibitor (CCR2 inhibitor, 1 μg/mL, Santa Cruz) for 30 min and allowed to migrate toward EVs in presence of CCR2 inhibitor. The cell movement were visualized and captured by Nikon Eclipse Ni-U microscope using 20X objective. The movement of cells was then analyzed using ImageJ 68 and Chemotaxis/migration tool (ibidi).

### Polarization of differentiated THP-1

THP-1 cells were purchased from Korean Cell Line Bank. THP-1 cells were maintained at 5 × 10^6^ cells/mL in RPMI (Gibco) supplemented with 10% heat-inactivated FBS (HyClone, GE healthcare Life Sciences) with 1% penicillin/streptomycin (Sigma-Aldrich). THP-1 cells were differentiated into M0 macrophages by treatment with PMA (100 ng/mL) for 48 h. M0-differentiated THP-1 cells (10^6^ cells) were exposed to NDTRs (10^8^ particles) and NDMVs (10^8^ particles) which were isolated from neutrophils (5 × 10^6^ cells) stimulated with fMLP (1 μM). For comparison, M0-differentiated THP-1 cells were stimulated with either LPS (100 ng/mL) and IFN-γ (10 ng/mL) for M1 polarization or IL-4 (10 ng/mL) and recombinant human IL-12 (10 ng/mL, Sinobiologicals) for M2 polarization. For evaluation of macrophage phenotype polarization, polarized-THP-1 cells were detached using trypsin supplemented with EDTA (Life Technologies), fixed with Phosflow buffer (BD biosciences), and further stained with fluorescence-labeled anti-human monoclonal antibodies: CD14 (PerCP, BD biosciences), HLA-DR (APC, BD biosciences), CD80 (PE, Bio Legend), CD86 (PE, Bio Legend), CD209 (APC, Bio Legend), CD23 (APC, Bio Legend), CD163 (PE, Bio Legend), and CD206 (FITC, BD biosciences). Flow cytometric analysis was made using BD FAC calibur and analyzed using FlowJo software. For evaluation of cytokine expressions, RNA was extracted from polarized-THP-1 cells using TRIzol (Ambion) and cDNA was synthesized using RT^2^ First strand kit according to manufacturer's recommended protocols (Qiagen). RT-qPCR was performed by using cDNA (100 ng), primer (1 μL) and RT^2^ SYBR Green qPCR mastermix (Qiagen) using Qiagen rotor. Briefly, reactions were performed under the suggested cycling conditions: polymerase activation for 10 min at 95 °C, followed by 40 cycles of 15 s at 95 °C and 45 s at 60 °C. For all PCR experiments, post-PCR DNA melt curve analysis was performed to assess amplification specificity. DNA melting was carried out using a temperature ramping rate of 1 °C per step with a 5-second rest at each step. Pre-designed RT^2^ qPCR primer assays (Qiagen) were used to determine the expression level of cytokines: iNOS (NOS2, PPH00173F), Arg-1 (PPH20977A), TNF-α (PPH00341F), TGF-β1 (PPH00508A), IL-10 (PPH00572C), IL-12A (PPH00544B), IL-6 (PPH00560C), and IL-1β (PPH00171C). Gene expression level were calculated as log_2_(2^-ΔΔct^) using GAPDH (PPH00150F) as a control. In transwell assay, bottom chamber was loaded with neutrophil-derived EVs and M0-differentiated THP-1 cells were cultured in upper chamber for 4 h. Then, THP-1 cells were harvested and subjected to qPCR.

### The uptake of neutrophil EVs by THP-1 cells

Neutrophils stained with cell tracker green (5 μg/mL, Invitrogen) were stimulated with fMLP and EVs were isolated. M0-differentiated THP-1 cells were exposed to neutrophil-derived EVs on a confocal dish and visualized using immunofluorescence microscopy at 37 °C, 5% CO_2_ for 1 h. For flow cytometric analysis, M0-differentiated THP-1 cells were exposed to EVs for indicated time. The cells were harvested, fixed with phosflow fix buffer, permeabilized with perm buffer, and stained with fluorescence-conjugated antibodies against CD14 (PE) and CD66b (FITC). The fluorescence levels of CD66b in CD14+ M0-differentiated THP-1 cells were examined. Sample acquisition was performed with BD FACS Calibur (BD Biosciences). The data was analyzed using FlowJo software.

### miRNA array and validation

Total RNA was isolated from neutrophil EVs using the miRNeasy Micro Kit (Qiagen, USA) following the manufacturer's protocol. The purity and concentration of the isolated RNA was measured with Nanodrop ND-1000 spectrophotometer (Nanodrop Technologies, USA). An equal amount (20 ng) of total RNA was converted to cDNA using the TaqMan MicroRNA Reverse Transcription Kit (Applied Biosystems, USA). 3 μL of RT product from total RNA was contained in a reaction volume of 10 μL with TaqMan Universal Master Mix II (Applied Biosystems) and TaqMan probe (hsa-miR-24-3p, hsa-miR-29a-3p, hsa-miR-122-5p, hsa-miR-126-3p, hsa-miR-150-5p, hsa-miR-451a, hsa-miR-1260a, hsa-miR-1285-5p, hsa-miR-4454, hsa-miR-7975) The expression levels of selected miRNAs were analyzed according to the TaqMan MicroRNA Assay protocol in triplicate. Cel-miR-39 was added in the samples during the RNA extraction step and was used as an exogenous control for normalization of raw data. The miRNA profile of the samples was analyzed with Nanostring nCounter^TM^ system. The effects of miRNAs (has-miR-1260a, hsa-miR-1285-5p, hsa-miR-4454, hsa-miR-7975, hsa-miR-126-3p, hsa-miR-150-5p, and hsa-miR-451a) on the phenotype polarization of macrophages were confirmed. M0-differentiated THP-1 (1 × 10^6^ cells) were transfected with 50 nM of indicated miRNA mimics (Applied Biological Materials) using NEPA21 (NEPA gene) and incubated for 24 h in RPMI. RNA was extracted and the expression of iNOS and Arginase were determined by quantitative PCR.

### Animal experiments

Animal experiments were approved by the Institutional Animal Care and Use Committee of Kyungpook National University and Hallym University. BALB/c (male, 5-8 weeks old) mice were purchased from Orient Bio. Bone marrow cells were isolated as previously described 69 and neutrophils were isolated using either neutrophil isolation kit (Miltenyi Biotec) or EasySep^TM^ mouse neutrophil enrichment kit (StemCell technologies) as previously described 38. EVs were separated by stimulating mouse neutrophils (5 × 10^6^ cells/mL) with *E. coli* (1 × 10^6^ cells/mL). EVs were dissolved in 100 μL RPMI and stored at -70 ℃. Experimental sepsis was induced in BALB/c mice by the cecal ligation and puncture (CLP) procedure as previously described Mice were treated with either NDTRs (10^8^ particles, 100 μL, i.p.), NDMVs (10^8^ particles, 100 μL, i.p.) or vehicle (saline,100 μL, i.p.) 30 min before CLP surgery, and further treated on day 1, 2, and 3 after CLP surgery. The survival of septic mice was monitored for 9 days. Peritoneal fluids were collected 18 h after CLP surgery, centrifuged at 1400 × g, and filtered through 1.2 μm filter. Supernatants and cells were subjected to enzyme-linked immunosorbent assay (ELISA) and macrophage isolation, respectively. The concentration of IL-1β, IL-6, and tumor necrosis factor (TNF)-α were measured using ELISA kit (R&D systems). Peritoneal macrophages were isolated using macrophage isolation kit (Miltenyi Biotec) and subjected to flow cytometric analysis. DSS-induced chronic colitis was performed according to previous study 39. In brief, BALB/c mice received water containing 2% DSS during first 5 days and further received normal drinking water for 10 days. At day 16, mice were exposed to water containing 2% DSS again until day 22. Mice were treated with NDTRs and NDMVs (10^8^ particles, 100 μL, i.p.) on days 16, 18, 20, and 22. Mice were sacrificed on day 22 and colon size were measured, and the colons were prepared for histological analysis as previously described 39.

### Statistical analysis

Data are presented as the mean ± SEM for continuous variables and as the number (%) for the categorical variables. Statistical data were analyzed by Graphpad prism 7.0e (GraphPad Software). Comparisons between two groups were performed with either two-tailed Student's t test (parametric) or Mann-Whitney (non-parametric test). Survival data were analyzed using Mantel-Cox log-rank test. Values of *p* < 0.05 were considered to indicate statistical significance.

## Summary

Neutrophils generate different types of extracellular vesicles. In this study, we found that neutrophil-derived trails are proinflammatory extracellular vesicles. Neutrophil-derived trails contained proinflammatory miRNAs, induced proinflammatory macrophage phenotype polarization, and showed protective effects in murine models of acute and chronic inflammation.

## Key points

- Neutrophil-derived trails contain proinflammatory miRNAs and induce proinflammatory macrophage polarization;
- Neutrophil-derived trails show protective effects in murine models of acute and chronic inflammation.

## Supplementary Material

Supplementary figures.

Supplementary tables.

Supplementary movie S1.

Supplementary movie S2.

Supplementary movie S3.

Supplementary movie S4.

## Abbreviations

EV: extracellular vesicle
NDMV: neutrophil-derived microvesicles
NDTR: neutrophil-derived trails
NK cells: natural killer cells
MoDCs: monocyte-derived dendritic cells
CXCL12: CXC-chemokine ligand 12
PAMPs: pathogen-associated molecular patterns
fMLP: formyl-methionyl-leucyl-phenylalanine
LPS: lipopolysaccharide
*E. coli*: *Escherichia coli*
*S. aureus*: *Staphylococcus aureus*
DAMPs: danger-associated molecular patterns
HMGB1: high mobility group box 1
TNF-α: tumor necrosis factor-α
IFN-γ: interferon-γ
TGF-β: tumor growth factor-β
IL-4: interleukin-4
L-NAME: N(γ)-nitro-L-arginine methyl ester
PMA: phorbol 12-myristate 13-acetate
MCP-1: monocyte chemoattractant protein 1
iNOS: inducible nitric oxide synthase

## References

[1] Thery C, Ostrowski M, Segura E (2009). Membrane vesicles as conveyors of immune responses. Nat Rev Immunol.

[2] van der Pol E, Boing AN, Gool EL, Nieuwland R (2016). Recent developments in the nomenclature, presence, isolation, detection and clinical impact of extracellular vesicles. J Thromb Haemost.

[3] Lim K, Hyun YM, Lambert-Emo K, Capece T, Bae S, Miller R (2015). Neutrophil trails guide influenza-specific CD8(+) T cells in the airways. Science.

[4] Hyun YM, Sumagin R, Sarangi PP, Lomakina E, Overstreet MG, Baker CM (2012). Uropod elongation is a common final step in leukocyte extravasation through inflamed vessels. J Exp Med.

[5] Gasser O, Hess C, Miot S, Deon C, Sanchez JC, Schifferli JA (2003). Characterisation and properties of ectosomes released by human polymorphonuclear neutrophils. Exp Cell Res.

[6] Dalli J, Montero-Melendez T, Norling LV, Yin X, Hinds C, Haskard D (2013). Heterogeneity in neutrophil microparticles reveals distinct proteome and functional properties. Mol Cell Proteomics.

[7] Eken C, Gasser O, Zenhaeusern G, Oehri I, Hess C, Schifferli JA (2008). Polymorphonuclear neutrophil-derived ectosomes interfere with the maturation of monocyte-derived dendritic cells. J Immunol.

[8] Eken C, Martin PJ, Sadallah S, Treves S, Schaller M, Schifferli JA (2010). Ectosomes released by polymorphonuclear neutrophils induce a MerTK-dependent anti-inflammatory pathway in macrophages. J Biol Chem.

[9] Eken C, Sadallah S, Martin PJ, Treves S, Schifferli JA (2013). Ectosomes of polymorphonuclear neutrophils activate multiple signaling pathways in macrophages. Immunobiology.

[10] Hess C, Sadallah S, Hefti A, Landmann R, Schifferli JA (1999). Ectosomes released by human neutrophils are specialized functional units. J Immunol.

[11] Mesri M, Altieri DC (1998). Endothelial cell activation by leukocyte microparticles. J Immunol.

[12] Mesri M, Altieri DC (1999). Leukocyte microparticles stimulate endothelial cell cytokine release and tissue factor induction in a JNK1 signaling pathway. J Biol Chem.

[13] Pliyev BK, Kalintseva MV, Abdulaeva SV, Yarygin KN, Savchenko VG (2014). Neutrophil microparticles modulate cytokine production by natural killer cells. Cytokine.

[14] Pluskota E, Woody NM, Szpak D, Ballantyne CM, Soloviev DA, Simon DI (2008). Expression, activation, and function of integrin alphaMbeta2 (Mac-1) on neutrophil-derived microparticles. Blood.

[15] Slater TW, Finkielsztein A, Mascarenhas LA, Mehl LC, Butin-Israeli V, Sumagin R (2017). Neutrophil Microparticles Deliver Active Myeloperoxidase to Injured Mucosa To Inhibit Epithelial Wound Healing. J Immunol.

[16] Timar CI, Lorincz AM, Csepanyi-Komi R, Valyi-Nagy A, Nagy G, Buzas EI (2013). Antibacterial effect of microvesicles released from human neutrophilic granulocytes. Blood.

[17] Headland SE, Jones HR, Norling LV, Kim A, Souza PR, Corsiero E (2015). Neutrophil-derived microvesicles enter cartilage and protect the joint in inflammatory arthritis. Sci Transl Med.

[18] Stein JM, Luzio JP (1991). Ectocytosis caused by sublytic autologous complement attack on human neutrophils. The sorting of endogenous plasma-membrane proteins and lipids into shed vesicles. Biochem J.

[19] Hong Y, Eleftheriou D, Hussain AA, Price-Kuehne FE, Savage CO, Jayne D (2012). Anti-neutrophil cytoplasmic antibodies stimulate release of neutrophil microparticles. J Am Soc Nephrol.

[20] Kambas K, Chrysanthopoulou A, Vassilopoulos D, Apostolidou E, Skendros P, Girod A (2014). Tissue factor expression in neutrophil extracellular traps and neutrophil derived microparticles in antineutrophil cytoplasmic antibody associated vasculitis may promote thromboinflammation and the thrombophilic state associated with the disease. Ann Rheum Dis.

[21] Dalli J, Norling LV, Renshaw D, Cooper D, Leung KY, Perretti M (2008). Annexin 1 mediates the rapid anti-inflammatory effects of neutrophil-derived microparticles. Blood.

[22] Duarte TA, Noronha-Dutra AA, Nery JS, Ribeiro SB, Pitanga TN, Lapa ESJR (2012). Mycobacterium tuberculosis-induced neutrophil ectosomes decrease macrophage activation. Tuberculosis (Edinb).

[23] Prakash PS, Caldwell CC, Lentsch AB, Pritts TA, Robinson BR (2012). Human microparticles generated during sepsis in patients with critical illness are neutrophil-derived and modulate the immune response. J Trauma Acute Care Surg.

[24] Gasser O, Schifferli JA (2004). Activated polymorphonuclear neutrophils disseminate anti-inflammatory microparticles by ectocytosis. Blood.

[25] Nolan S, Dixon R, Norman K, Hellewell P, Ridger V (2008). Nitric oxide regulates neutrophil migration through microparticle formation. Am J Pathol.

[26] Pitanga TN, de Aragao Franca L, Rocha VC, Meirelles T, Borges VM, Goncalves MS (2014). Neutrophil-derived microparticles induce myeloperoxidase-mediated damage of vascular endothelial cells. BMC Cell Biol.

[27] Soehnlein O, Steffens S, Hidalgo A, Weber C (2017). Neutrophils as protagonists and targets in chronic inflammation. Nat Rev Immunol.

[28] Rhys HI, Dell'Accio F, Pitzalis C, Moore A, Norling LV, Perretti M (2018). Neutrophil Microvesicles from Healthy Control and Rheumatoid Arthritis Patients Prevent the Inflammatory Activation of Macrophages. EBioMedicine.

[29] Johnson BL 3rd, Midura EF, Prakash PS, Rice TC, Kunz N, Kalies K (2017). Neutrophil derived microparticles increase mortality and the counter-inflammatory response in a murine model of sepsis. Biochim Biophys Acta Mol Basis Dis.

[30] Nauseef WM, Borregaard N (2014). Neutrophils at work. Nat Immunol.

[31] Thery C, Witwer KW, Aikawa E, Alcaraz MJ, Anderson JD, Andriantsitohaina R (2018). Minimal information for studies of extracellular vesicles 2018 (MISEV2018): a position statement of the International Society for Extracellular Vesicles and update of the MISEV2014 guidelines. J Extracell Vesicles.

[32] Camussi G, Deregibus MC, Bruno S, Cantaluppi V, Biancone L (2010). Exosomes/microvesicles as a mechanism of cell-to-cell communication. Kidney Int.

[33] Valadi H, Ekstrom K, Bossios A, Sjostrand M, Lee JJ, Lotvall JO (2007). Exosome-mediated transfer of mRNAs and microRNAs is a novel mechanism of genetic exchange between cells. Nat Cell Biol.

[34] van der Pol E, Boing AN, Harrison P, Sturk A, Nieuwland R (2012). Classification, functions, and clinical relevance of extracellular vesicles. Pharmacol Rev.

[35] Graff JW, Dickson AM, Clay G, McCaffrey AP, Wilson ME (2012). Identifying functional microRNAs in macrophages with polarized phenotypes. J Biol Chem.

[36] O'Connell RM, Rao DS, Chaudhuri AA, Baltimore D (2010). Physiological and pathological roles for microRNAs in the immune system. Nat Rev Immunol.

[37] Saha B, Momen-Heravi F, Kodys K, Szabo G (2016). MicroRNA Cargo of Extracellular Vesicles from Alcohol-exposed Monocytes Signals Naive Monocytes to Differentiate into M2 Macrophages. J Biol Chem.

[38] Park SY, Shrestha S, Youn YJ, Kim JK, Kim SY, Kim HJ (2017). Autophagy Primes Neutrophils for Neutrophil Extracellular Trap Formation during Sepsis. Am J Respir Crit Care Med.

[39] Marcon R, Bento AF, Dutra RC, Bicca MA, Leite DF, Calixto JB (2013). Maresin 1, a proresolving lipid mediator derived from omega-3 polyunsaturated fatty acids, exerts protective actions in murine models of colitis. J Immunol.

[40] Raeven P, Zipperle J, Drechsler S (2018). Extracellular Vesicles as Markers and Mediators in Sepsis. Theranostics.

[41] van de Winkel JG, Capel PJ (1993). Human IgG Fc receptor heterogeneity: molecular aspects and clinical implications. Immunol Today.

[42] Seveau S, Eddy RJ, Maxfield FR, Pierini LM (2001). Cytoskeleton-dependent membrane domain segregation during neutrophil polarization. Mol Biol Cell.

[43] Rossy J, Schlicht D, Engelhardt B, Niggli V (2009). Flotillins interact with PSGL-1 in neutrophils and, upon stimulation, rapidly organize into membrane domains subsequently accumulating in the uropod. PLoS One.

[44] Derrick T, Ramadhani AM, Mtengai K, Massae P, Burton MJ, Holland MJ (2017). miRNAs that associate with conjunctival inflammation and ocular Chlamydia trachomatis infection do not predict progressive disease. Pathog Dis.

[45] Derrick T, Roberts C, Rajasekhar M, Burr SE, Joof H, Makalo P (2013). Conjunctival MicroRNA expression in inflammatory trachomatous scarring. PLoS Negl Trop Dis.

[46] Meng QL, Liu F, Yang XY, Liu XM, Zhang X, Zhang C (2014). Identification of latent tuberculosis infection-related microRNAs in human U937 macrophages expressing Mycobacterium tuberculosis Hsp16.3. BMC Microbiol.

[47] Fabbri M, Paone A, Calore F, Galli R, Gaudio E, Santhanam R (2012). MicroRNAs bind to Toll-like receptors to induce prometastatic inflammatory response. Proc Natl Acad Sci U S A.

[48] Momen-Heravi F, Bala S, Kodys K, Szabo G (2015). Exosomes derived from alcohol-treated hepatocytes horizontally transfer liver specific miRNA-122 and sensitize monocytes to LPS. Sci Rep.

[49] Fell LH, Seiler-Mussler S, Sellier AB, Rotter B, Winter P, Sester M (2016). Impact of individual intravenous iron preparations on the differentiation of monocytes towards macrophages and dendritic cells. Nephrol Dial Transplant.

[50] Armstrong DA, Nymon AB, Ringelberg CS, Lesseur C, Hazlett HF, Howard L (2017). Pulmonary microRNA profiling: implications in upper lobe predominant lung disease. Clin Epigenetics.

[51] Cobos Jimenez V, Bradley EJ, Willemsen AM, van Kampen AH, Baas F, Kootstra NA (2014). Next-generation sequencing of microRNAs uncovers expression signatures in polarized macrophages. Physiol Genomics.

[52] Escate R, Padro T, Badimon L (2016). LDL accelerates monocyte to macrophage differentiation: Effects on adhesion and anoikis. Atherosclerosis.

[53] Harris TA, Yamakuchi M, Ferlito M, Mendell JT, Lowenstein CJ (2008). MicroRNA-126 regulates endothelial expression of vascular cell adhesion molecule 1. Proc Natl Acad Sci U S A.

[54] Lin JB, Moolani HV, Sene A, Sidhu R, Kell P, Lin JB (2018). Macrophage microRNA-150 promotes pathological angiogenesis as seen in age-related macular degeneration. JCI Insight.

[55] Manoharan P, Basford JE, Pilcher-Roberts R, Neumann J, Hui DY, Lingrel JB (2014). Reduced levels of microRNAs miR-124a and miR-150 are associated with increased proinflammatory mediator expression in Kruppel-like factor 2 (KLF2)-deficient macrophages. J Biol Chem.

[56] Shantikumar S, Caporali A, Emanueli C (2012). Role of microRNAs in diabetes and its cardiovascular complications. Cardiovasc Res.

[57] Dalli J, Norling LV, Montero-Melendez T, Federici Canova D, Lashin H, Pavlov AM (2014). Microparticle alpha-2-macroglobulin enhances pro-resolving responses and promotes survival in sepsis. EMBO Mol Med.

[58] Amulic B, Cazalet C, Hayes GL, Metzler KD, Zychlinsky A (2012). Neutrophil function: from mechanisms to disease. Annu Rev Immunol.

[59] Nauseef WM (2016). Neutrophils, from cradle to grave and beyond. Immunol Rev.

[60] Jones HR, Robb CT, Perretti M, Rossi AG (2016). The role of neutrophils in inflammation resolution. Semin Immunol.

[61] van der Poll T, van de Veerdonk FL, Scicluna BP, Netea MG (2017). The immunopathology of sepsis and potential therapeutic targets. Nat Rev Immunol.

[62] Yang X, Yin Y, Yan X, Yu Z, Liu Y, Cao J (2019). Flagellin attenuates experimental sepsis in a macrophage-dependent manner. Crit Care.

[63] Carestia A, Mena HA, Olexen CM, Ortiz Wilczynski JM, Negrotto S, Errasti AE (2019). Platelets Promote Macrophage Polarization toward Pro-inflammatory Phenotype and Increase Survival of Septic Mice. Cell Rep.

[64] Genschmer KR, Russell DW, Lal C, Szul T, Bratcher PE, Noerager BD (2019). Activated PMN Exosomes: Pathogenic Entities Causing Matrix Destruction and Disease in the Lung. Cell.

[65] Hong CW, Kim TK, Ham HY, Nam JS, Kim YH, Zheng H (2010). Lysophosphatidylcholine increases neutrophil bactericidal activity by enhancement of azurophil granule-phagosome fusion via glycine.GlyR alpha 2/TRPM2/p38 MAPK signaling. J Immunol.

[66] Zhou J, Ghoroghi S, Benito-Martin A, Wu H, Unachukwu UJ, Einbond LS (2016). Characterization of Induced Pluripotent Stem Cell Microvesicle Genesis, Morphology and Pluripotent Content. Sci Rep.

[67] Repnik U, Knezevic M, Jeras M (2003). Simple and cost-effective isolation of monocytes from buffy coats. J Immunol Methods.

[68] Schneider CA, Rasband WS, Eliceiri KW (2012). NIH Image to ImageJ: 25 years of image analysis. Nat Methods.

[69] Swamydas M, Lionakis MS Isolation, purification and labeling of mouse bone marrow neutrophils for functional studies and adoptive transfer experiments. J Vis Exp. 2013: e50586.

