# Supplementary material for: Neutrophil-derived trail is a proinflammatory subtype of neutrophil-derived extracellular vesicles
Source: Theranostics. 2021 Jan 1;11(6):2770–87. doi: 10.7150/thno.51756 (PMC7806483; doi:10.7150/thno.51756)
Supplement: Supplementary file 1 — Supplementary figures. [file thnov11p2770s1.pdf]

Supplementary methods

Animal experiments

For cecal ligation and puncture, BALB/c mice were anesthetized with pentobarbital (50 mg/kg, i.p.), and an abdominal mid-line incision was made. After laparotomy, the cecum was ligated below ileocecal valve, punctured with 22-gauge needle at two sides, and abdomen was closed. For histological analysis, colons from mouse with DSS-induced colitis were fixed with 4% formaldehyde solution (Biosesang) and embedded in paraffin. Further, paraffin embedded samples were sectioned at 5 μm-thickness and attached to adhesive glass slides (Matsunami), and stained with hematoxylin (Dako Mayer's Hematoxylin, Agilent) and eosin (Daejung chemical and metal Co Ltd.). The HE stained tissues were visualized using Nikon Eclipse Ni microscope at 10 × objective. For histological analysis, mononuclear cells and polymorphonuclear cell infiltration in lamina propria, crypt inflammation, epithelial hyperplasia, and erythrocyte loss were scored.

Calibration between calcein fluorescence and particle concentrations of neutrophil-derived EVs

Neutrophils (5×10^8 cells) were stained with calcein-AM (20 μg/mL, Merck Millipore) and EVs were separated. EVs were diluted in 1 × PBS at indicated ratio (1:5, 1:10, and 1:50). The calcein-fluorescence of EVs was measured using Spectramax M2/e fluorescence microplate reader (Molecular Devices) and concentrations of EVs was measured using Nanosight LM10.

The inhibition of EV generation from neutrophils

For inhibition of neutrophil EVs generation, calcein-stained neutrophils were stimulated with fMLP (1 μM, Sigma-Aldrich), PMA (100 μg/mL, Sigma-Aldrich), C5a (50 ng/mL, Sino Biologicals) and TNF-α (50 ng/mL, Sino Biologicals) in presence of various inhibitors; inhibitor of ERK MAP kinase (PD90859, 10 μM, Tocris bioscience), inhibitor of p38 MAP kinase (SB203580, 10 μM, Tocris bioscience), calcium chelator (BAPTA-AM, 10 μM, Tocris bioscience), inhibitor of calcium-activated chloride channel (CaCCinh-A01, 30 ng/mL, Sigma-Aldrich), inhibitor of actin polymerization (Cytochalasin D, 50 μg/mL, Sigma-Aldrich), inhibitor of PI3-Kinase (Wortmannin, 100 nM, Tocris bioscience), inhibitor of Rho-A GTPase (Tat C3, 20 ng/mL, provided from Prof. Park in Hallym University), inhibitor of Rac-1 GTPase (NSC23766, 10 μM, Tocris bioscience), inhibitor of cdc42 (ML141, 10 μM, Tocris bioscience), inhibitor of β1 integrin very late antigen (VLA)-4 (Bio1211, 10 μM, Tocris bioscience), inhibitor of macrophage-1 (MAC-1) antigen (IMB10, 10 μM, Sigma-Aldrich), inhibitor of mTOR pathway (Rapamycin, 10 nM, Sigma-Aldrich), inhibitors on the autophagic flux pathways (3-MA, 5 μM, Sigma-Aldrich; chloroquine, 5 μM, Sigma-Aldrich; bafilomycin, 5 μM, Sigma-Aldrich). EVs were isolated and calcein fluorescence was measured using spectrofluorometer.

Flow cytometric analysis of NDTRs

The expressions of surface markers on NDTRs were analyzed using flow cytometry in accordance with previous study (1). Isolated NDTRs and NDMVs were fixed with Phosflow buffer (BD biosciences) and stained with fluorescence tagged anti-human antibodies for 1 h at 4 °C. For surface marker evaluation, neutrophil EVs were stained with Annexin A5 (FITC, Abcam) or FITC-conjugated antibody against Annexin A1 (Annexin A1, Bio Legend; FITC conjugate, Abcam), CD9 (FITC, Bio Legend), CD81 (FITC, Bio Legend), CD63 (FITC, BD biosciences), CD66b

(FITC, BD biosciences), CD35 (PE, BD biosciences), CD11b (FITC, Invitrogen), CD49d (FITC, Bio Legend), CD29 (PE, eBioscience), CD18 (PE, eBioscience), CD43 (FITC, Bio Legend), PSGL-1 (FITC, Abcam), flotillin-2 (PE, Abcam), and CD16 (PE, BD biosciences). For evaluation of contents, fixed EVs were permeabilized with phosflow perm buffer (BD biosciences), and stained with MCP (FITC, eBioscience) and HSP (APC, Invitrogen). Since size of neutrophil-derived EVs are smaller than the wavelength of laser used, false trigger from background restricted the measurement of sizes of neutrophil-derived EVs. Forward and side light scatters are nonspecific trigger parameters owing to smaller size of neutrophil-derived EVs and the difference in the refractive index of beads and neutrophil-derived EVs. Therefore, 1 μm beads (Life Technologies) was employed as an indirect calibrator of light scatter for neutrophil-derived EV size required for gating strategy. Then, we used fluorescence trigger as described by Nolan et al. to detect neutrophil-derived EVs. The samples were acquired using BD FACS calibur (BD Biosciences) at high speed (60 ± 7 μL/min) for 60 sec. Detection was triggered using blue laser (488 nm) and red diode laser (635 nm), excited green fluorescence (530 nm/30 nm bandpass), along with other fluorescence parameters were recorded in log forward light scatter (FSC) and log side light scatter (SSC). Neutrophil-derived EV population were gated between 1 μM beads and the events recorded using buffer only, then the fluorescence histogram was prepared. To confirm that fluorescence is specific to membrane staining of neutrophil-derived EVs, aliquot of EVs were lysed by addition of sodium dodecyl sulfate [SDS, 0.025% (w/w)] as previously described (2). Specifically, EVs were lysed with 0.025% SDS, vortexed for 1 min, centrifuged at 14,000g for 10 min, and supernatants were removed. The pellets were washed twice with ice-chilled 1 × PBS and resuspended. The lysis of neutrophil-derived EVs was confirmed using NTA. Untouched EVs, EVs stained with annexin A5, lysed annexin A5-stained EVs by detergent, and annexin A5 without

EVs were analyzed using flow cytometry. The data was analyzed using FlowJo software (TreeStar Inc.).

Electrophoresis

DNA was extracted from neutrophil-derived EVs (10^{10} particles) isolated from neutrophils (5×10^8) using DNA extraction kit (QIAamp DNA Mini Kit, Qiagen) according to manufacturer's instruction. The extracted DNA was separated in 0.8% agarose gel and visualized with UV-trans illuminator. Total RNA was extracted using TRIZOL method, separated in 0.8% agarose gel and visualized in a UV-trans illuminator. For determination of protein, EVs (10^{10} particles) isolated from neutrophils (5×10^8) were lysed using RIPA lysis buffer. The lysate was separated by SDS-PAGE under reducing conditions and visualized using coomassie blue staining (Coomassie brilliant blue R250, Bio Rad).

ROS generation

Neutrophil derived EVs (2×10^8 particles) derived from fMLP-stimulated neutrophils (10^7 cells) were treated with PMA (1 μg/mL) followed by addition of horseradish peroxidase (2 mg/mL, Sigma-Aldrich) and luminol (2 mg/mL, Sigma-Aldrich) in a Hank's balanced salt solution. Kinetics was measured using spectramax i3X (Molecular devices) for 2400 sec at an interval of 45 sec. To confirm ROS generation, superoxide dismutase (SOD) inhibition activity and cytochrome c reduction assay was performed using superoxide dismutase activity assay kit (Abcam) and cytochrome c Reductase (NADPH) assay kit (Sigma-Aldrich) following manufacturer's instruction.

Chemotaxis assay of neutrophils

Migration of neutrophils against NDMV and NDTR were determined using μ-slide chamber (ibidi) following manufacturer's instruction. Neutrophils were allowed to migrate toward direct NDTRs, isolated NDTRs, and isolated NDMVs. For inhibition of neutrophil chemotaxis, neutrophils were pre-treated with LY223982 (leukotriene B4 antagonist, 10 μg/mL, Santa Cruz) for 30 min and allowed to migrate against EVs in presence of LY223982. The cell movement were visualized and captured by Nikon Eclipse Ni-U microscope using 20 × objective. The movement of cells was then analyzed using ImageJ and Chemotaxis/migration tool (ibidi).

Polarization of M0-differentiated THP-1 cells

THP-1 cells (Korean Cell Line Bank) were differentiated into M0 macrophages by treatment with PMA (100 ng/mL). M0-differentiated THP-1 cells (10^6 cells) were further exposed to neutrophil EVs (10^8 particles) in presence of annexin A5, anti-annexin A1, lysed EVs with detergent (0.025% SDS), or filtrate from EV-suspended media. Filtrate from EV-suspended media was collected by filtration of supernatants from EV-suspended media through 50 μm hydrophilic syringe filter (TISCH Scientific, USA). To investigate the requirement of physical contact in the effects of neutrophil-derived EVs, M0-differentiated THP-1 cells were exposed to neutrophil-derived EVs in presence of anti-annexin A5 antibody (+AnxA5) and anti-annexin A1 antibody (+AnxA1). For transwell experiment, neutrophil-derived EVs were loaded in the bottom chamber of transwell (Corning® HTS transwell®-24 well, 0.4 μm pore polycarbonate membrane) and M0-differentiated THP-1 cells were loaded in the upper chamber. After 4 h, THP-1 cells were harvested and the

expression of iNOS and arginase were examined using qPCR. RNA was extracted from differentiated THP-1 after 24 h using TRIZOL and cDNA was synthesized using RT^2 First strand kit (Qiagen). Pre-designed RT^2 qPCR primer assays (Qiagen) were used to determine the expression level of iNOS and Arg-1: iNOS (NOS2, PPH00173F), Arg-1 (PPH20977A).

Bactericidal activity

Opsonized *E. coli* (DH5α, 10^7 cells) and *S. aureus* (ATCC 25923, 10^7 cells) were exposed to neutrophil-derived EVs (2×10^9 particles) isolated from neutrophils (10^8 cells) stimulated with respective bacteria. To confirm the effect of neutrophil-derived EVs, bacteria were exposed to either lysed EVs or filtrated EVs. Then, bacteria were incubated in LB broth for 30 min in a 1.5 ml Eppendorf tube at 37 °C with 5% CO_2. Then, bacteria were centrifuged, washed with 1 × PBS, and incubated with ice-chilled DDW for 30 min. Serial dilutions of the homogenates were plated on LB agars and the bacteria CFUs (colony forming units) were counted after 24 h.

Nano LC-MS/MS analysis

Proteins were extracted from isolated NDMVs and NDTRs using 1 × RIPA lysis buffer (Bio basics Canada Inc.) containing protease inhibitor cocktail (ROCHE) and phosphatase inhibitor cocktail (GenDEPOT). Then in-solution tryptic digestion was performed, followed by Nano LC-MS/MS analysis with a nano HPLC system (Agilent, Wilmington, DE). The nano chip column (Agilent, Wilmington, DE, 150 mm × 0.075 mm) was used for peptide separation. The mobile phase A for LC separation was 0.1% formic acid in deionized water and the mobile phase B was 0.1% formic acid in acetonitrile. The chromatography gradient was designed for a linear increase from 3% B to

45% B in 70 min, 45% B to 95% B in 1 min, 95% B in 9 min, and 3% B in 10 min. The flow rate was maintained at 300 nL/min. Product ion spectra were collected in the information-dependent acquisition (IDA) mode and were analyzed by Agilent 6530 Accurate-Mass Q-TOF using continuous cycles of one full scan TOF MS from 300-2000 *m*/*z* (1.0 s) plus three product ion scans from 150-2000 *m*/*z* (1.5 s each). Precursor *m*/*z* values were selected starting with the most intense ion, using a selection quadrupole resolution of 3 Da. The rolling collision energy feature was used, which determines collision energy based on the precursor value and charge state. The dynamic exclusion time for precursor ion *m*/*z* values was 60 s.

Database searching and Gene ontology Enrichment analysis

Spectra were searched against SwissProt human database (20,317 sequences) using the R 3.6, Bioconductor 3.10, R for Proteomics, MSGFPlus package [4][5]. Trypsin enzyme parameter was selected with maximum two cleavages. The following additional search parameters were used: carbamidomethylation on Cys as fixed modification, acetylation N-terminal protein and oxidation on Met as variable modification, parent ion tolerance of 20 ppm, and peptide length range from 6 to 25 for peptide identification. Further, the identified proteins (363) were confirmed using vesiclepedia data, total proteins (239) that were common with vesiclepedia data were used for gene ontology analysis (GO) to identify biological processes, molecular functions and cellular components using PANTHER overrepresentation test, GO enrichment analysis.

Serum evaluation

Serum obtained from sepsis patients who admitted to the intensive care unit of Kyungpook

National University Hospital between April and October 2016. Adults patients (age ≥ 18) with sepsis in community-acquired pneumonia were enrolled. All patients provided informed consents in accordance with the Declaration of Helsinki. Venous blood was taken from septic patients within 24 h after ICU admission. Serum were obtained before neutrophil isolation and further stored at -70 ℃. Further, serum was thawed and filtered through 1.2 μM filter. The filtrate was then treated with 5μl of ExoQuick (System Biosciences), incubated for 30 mins at 4 ℃, followed by high-speed centrifugation (14000 × for 1 h). The pellets were then fixed with Phsoflow fix buffer and stained with annexin A5 antibody (FITC), anti-CD16 antibody (PE), and anti-CD66b antibody (PerCP). The sample were then acquired using BD FACS Calibur and the data analyzed using FlowJo. Annexin A5 positive subset was considered as EVs. EVs were further gated according to CD66b expression and the expression level of CD16 was employed to discriminate NDTRs and NDMVs. The percentages of NDEVs was calculated as: NDTRs (%) = (particle numbers of $AnxA_5^+$ $CD66b^+$ $CD16^-$ subset)/(particle numbers of $AnxA_5^+$ subset) × 100), NDMVs (%) = (particle numbers of $AnxA_5^+$ $CD66b^+$ $CD16^+$subset)/(particle numbers of $AnxA_5^+$ subset) × 100).

References

1. J. P. Nolan, E. Duggan, Analysis of Individual Extracellular Vesicles by Flow Cytometry. *Methods Mol Biol* **1678**, 79-92 (2018).
2. X. Osteikoetxea *et al.*, Differential detergent sensitivity of extracellular vesicle subpopulations. *Org Biomol Chem* **13**, 9775-9782 (2015).
3. L G. Companion package to the 'Using R and Bioconductor for proteomics data analysis' publication. *R package version 1.24.0*. 2019
4. TL P. MSGFplus: An interface between R and MS-GF+ . *R package version 1.20.0*. 2019.

1

Supplementary movie legends

Movie S1. Related to Figure 1A. Live imaging of NDTR generation from neutrophils. Neutrophils stained with call tracker were incubated with μ-slide chamber pre-coated with fibronectin in presence with various stimulators.

Movie S2. Related to Figure 1A. Live imaging of NDMV generation from neutrophils. Neutrophils stained with call tracker were incubated with confocal plates in presence with various stimulators.

Movie S3. Related to Figure 4A. Live imaging of NDTR uptake by M0-differentiated THP-1 cells. NDTRs were stained with calcein-AM

Movie S4. Related to Figure 4A. Live imaging of NDMV uptake by M0-differentiated THP-1 cells. NDMVs were stained with calcein-AM

Supplementary figures and legends

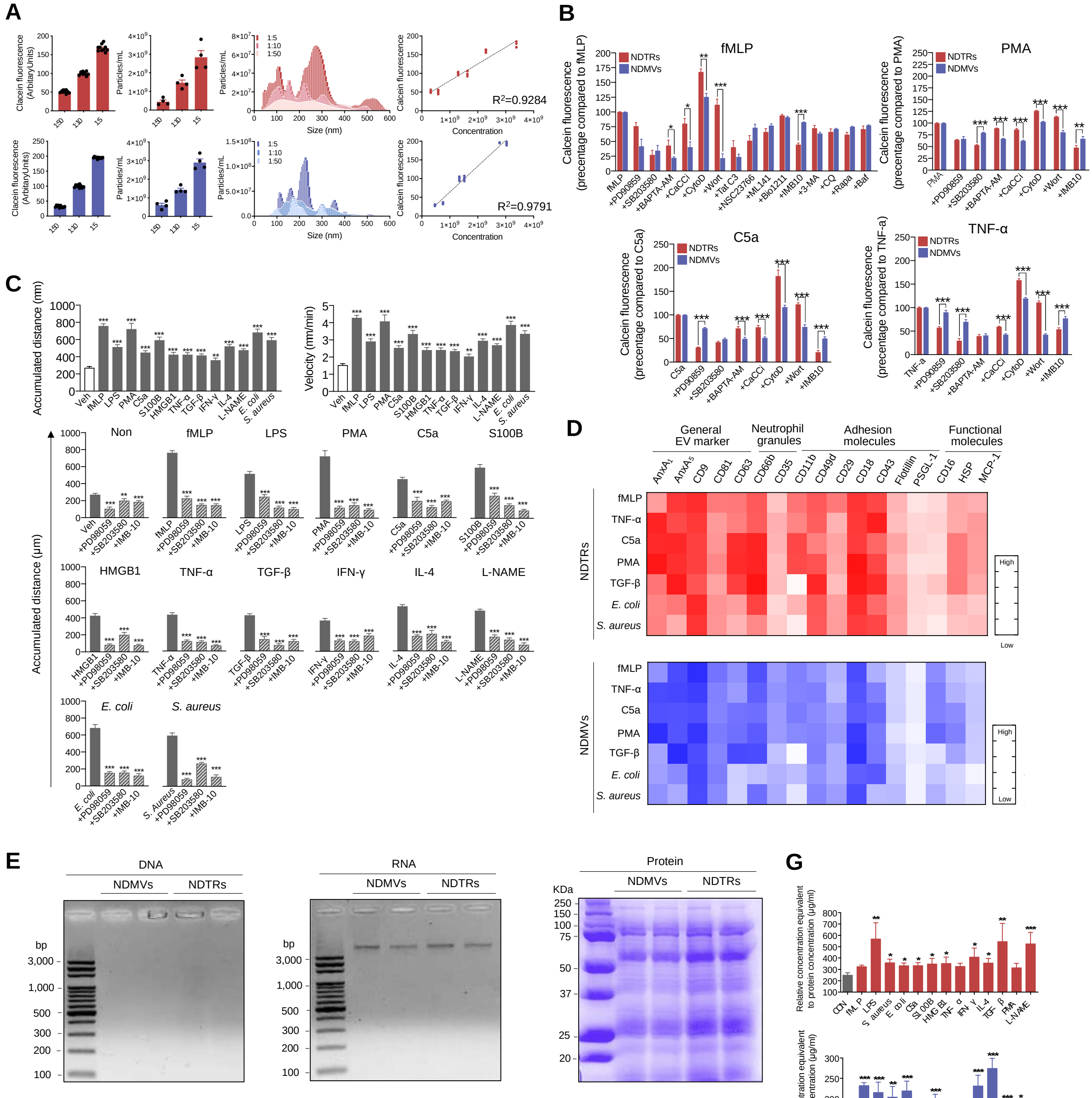

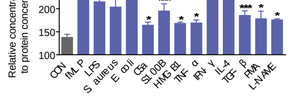

F

EV	Diameter (nm) [mean ± SEM, (median± SEM)]	Concentration (10^8 Particles/mL) (mean ± SEM)	DNA (ng/μL)	RNA (ng/μL) (mean ± SEM)	Protein (ng/μL) (mean ± SEM)
NDTRs	232.1 ± 16.25 (220.3 ± 12.21)	12.62 ± 1.45	N.D.	98.47 ± 0.27	21.28 ± 2.99
NDMVs	227.2 ± 9.324 (196.7 ± 7.69)	14.63 ± 1.82	N.D.	92.27 ± 1.57	46.95 ± 3.02

*Composition of NDTRs and NDMVs from neutrophils (5 × 10^7 cells) stimulated with fMLP

Figure S1. Related to Figure 1. Phenotype characterization of NDTRs (A) The quantification of neutrophil-derived EVs. NDTRs and NDMVs were isolated from neutrophils stained with

calcein-AM. Neutrophils were stimulated with fMLP (1 μM) and EVs were separated. Separated EVs were aliquoted, diluted as indicated (1:5, 1:10, and 1:50), and quantified using spectrofluorometer and NTA (n = 3 per each group). Left panels, Relative calcein fluorescence intensity of neutrophil-derived EVs; Middle panels, Size distribution and concentration of neutrophil-derived EVs using NTA; Right panels, the positive correlation between relative calcein-fluorescence intensities and particle numbers. All data are representative of three independent experiments. (B) The effects of signaling pathway inhibitors on the EV formations from neutrophils. Neutrophils were stained with calcein-AM and further stimulated with fMLP (1 μM), PMA (100 μg/mL), C5a (50 ng/mL), and TNF-α (50 ng/mL) in the presence or absence of indicated inhibitors. Neutrophil-derived EVs were separated and calcein fluorescence was measured using spectrofluorometer. n = 3 per each group. (C) Chemokinesis of neutrophils in response to various stimulators. Neutrophils were allowed to move against indicated stimulators in chemotaxis chamber in the presence of absence of indicated inhibitors. Bar graphs denote accumulated distance and mean velocity of neutrophils traveled toward indicated stimulators. (D) Heatmap depicting marker expression levels in NDTRs and NDMVs. EVs were assessed for the expressions of various markers using flow cytometric analysis. (E-F) Quantification of contents in neutrophil-derived EVs. EVs were separated from fMLP-stimulated neutrophils. DNA, RNA, and proteins were extracted, and concentrations were measured using Nanodrop (DNA, RNA) and Bradford method (Protein). (F) Summarization of sizes, concentrations, and contents of neutrophil-derived EVs. (G) Quantification of protein concentrations in neutrophil-derived EVs. Calcein-stained neutrophils were allowed to generate EVs in response to indicated stimulators. The fluorescence in EVs was calculated to equivalent protein concentration. n = 3. All data shown as mean ± SEM. *P < 0.05; **P < 0.001; ***P < 0.0001.

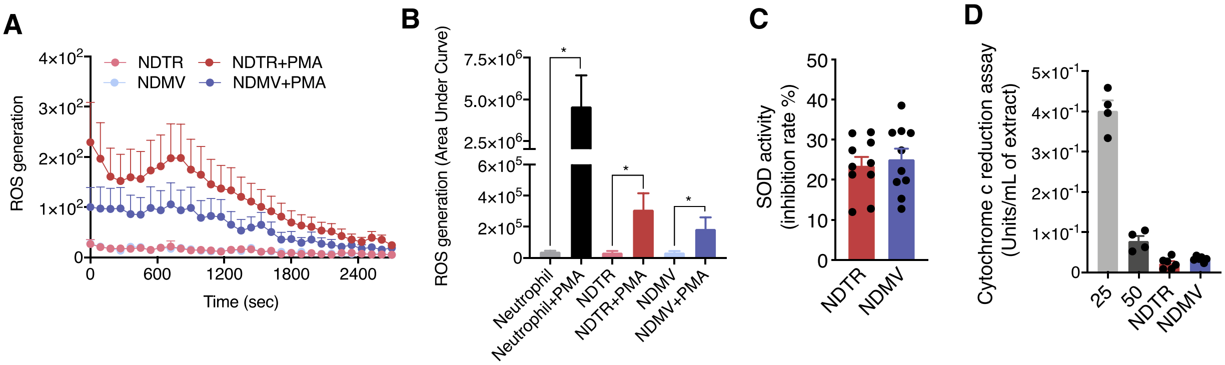

Figure S2. Related to Figure 2. ROS generation from neutrophil-derived EVs. (A-B) Luminol assay of neutrophil-derived EVs. (A) Either neutrophils or neutrophil-derived EVs were stimulated with PMA (100 μg/mL) and followed by addition of horseradish peroxidase and luminol. The kinetics of ROS generation was measured for 40 min at an interval of 45 sec. n = 6 per each group. (B) Area under the curve of ROS generation. (C) SOD activity of neutrophil-derived EVs. n = 10. (D) Cytochrome reductase assay of neutrophil-derived EVs compared to 1:25 (25) and 1:50 (50) diluted positive control. n = 3-6 per each group. Data are shown as the mean ± SEM. *P < 0.05.

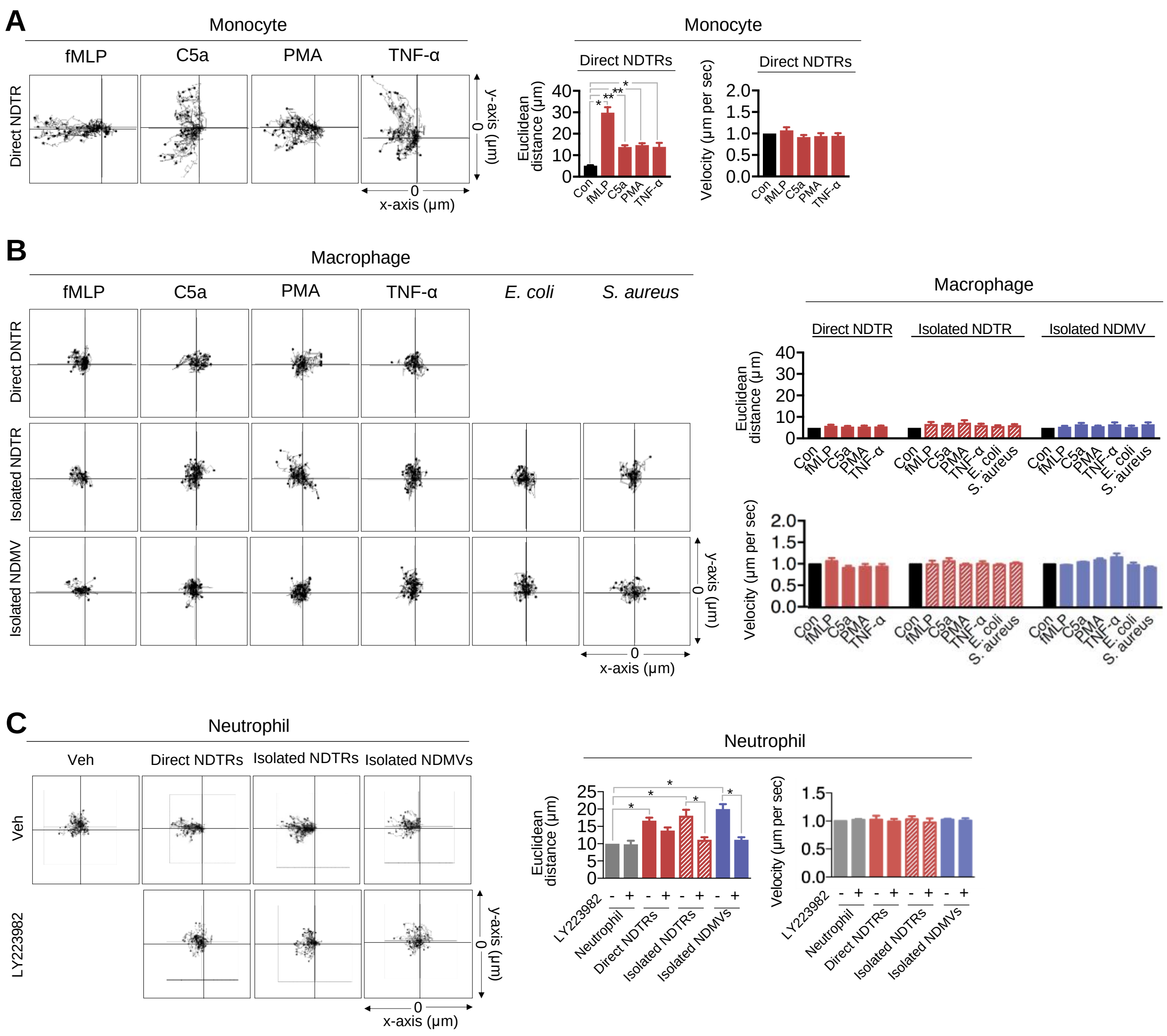

Figure S3. Related to Figure 3. Chemotaxis of macrophages and neutrophils against neutrophil-derived EVs. (A-C) Tracking analysis on chemotaxis of monocytes, macrophages, and neutrophils against neutrophil-derived EVs. The distances of migrated cells were tracked on every minute for 45 min. Direct NDTR: neutrophils were allowed to generate NDTRs according to indicated stimulators on a fibronectin-coated μ-slide chamber and target cells were allowed to move directly toward NDTRs. Isolated NDTRs and NDMVs, EVs were separated from neutrophils stimulated with indicated stimulators and were loaded to the lane of μ-slide chamber. Target cells were allowed to move toward neutrophil-derived EVs. The representative tracking results of thirty

cells per each group were presented. Bar graph denotes mean distance and velocity of target cells (A) The chemotaxis of monocytes against direct NDTRs. (B) The chemotaxis of macrophages against neutrophil-derived EVs. (C) The chemotaxis of neutrophils against neutrophil-derived EVs. LY223982, neutrophils were loaded in the presence of BLT_1 receptor antagonist. Data are shown as the mean ± SEM. *P < 0.05; **P < 0.01; ***P < 0.001.

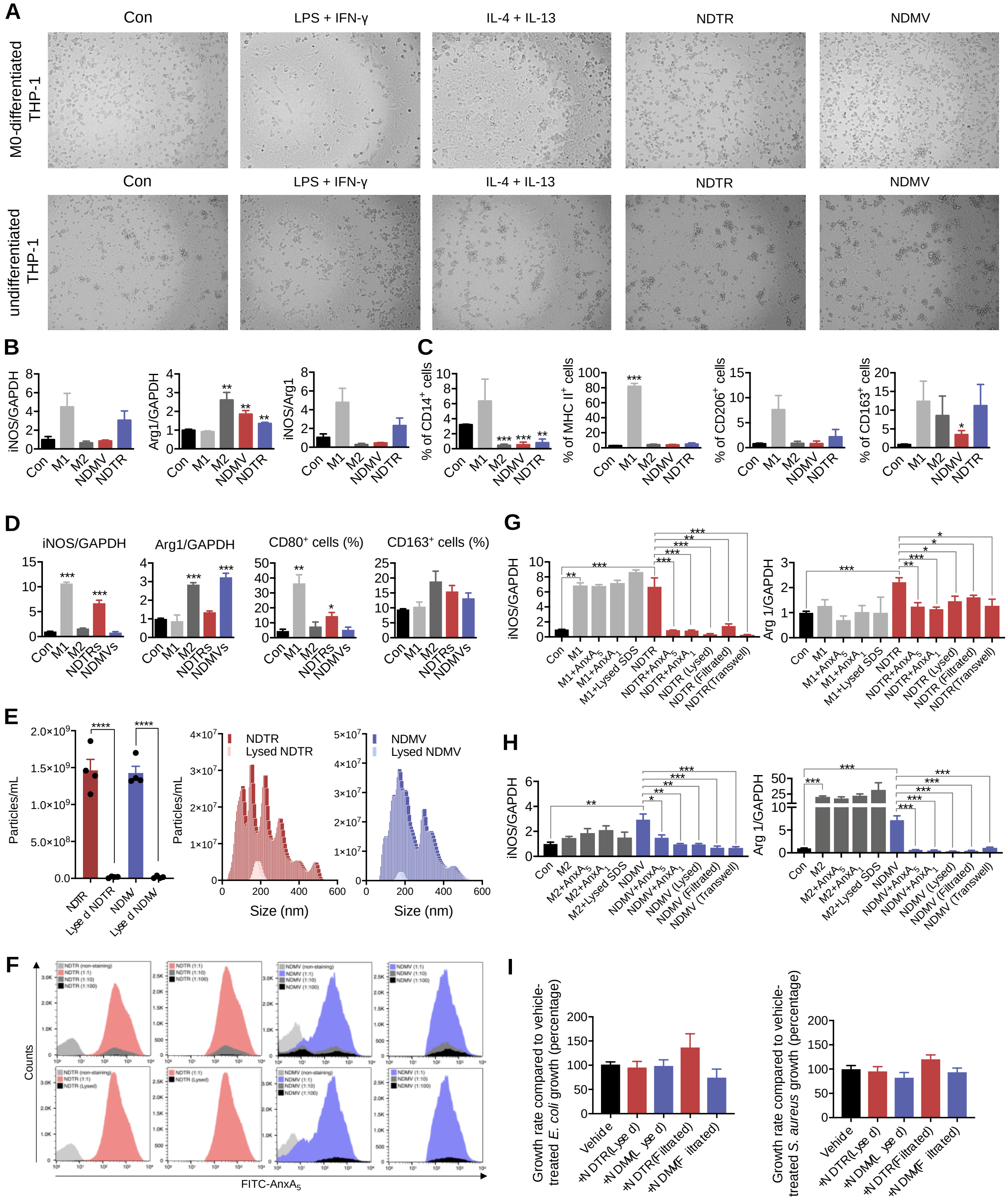

Figure S4. Related Figure 4. The effects of NDTRs on undifferentiated THP-1 cells and evaluation of effector mechanisms of NDTRs (A-C) Effects of neutrophil-derived EVs on

phenotype polarization of undifferentiated THP-1 cells. (A) Representative morphology of M0-differentiated and undifferentiated THP-1 cells. (B) The fold change in cytokines expression levels of M1 and M2 macrophage-associated markers in undifferentiated cells exposed to neutrophil-derived EVs. n = 3 per each group. (C) The surface marker expression of M1 and M2 macrophage-associated markers in undifferentiated cells exposed to neutrophil-derived EVs (n = 3 per each group). (F) The expression levels of phenotype markers in monocytes exposed to neutrophil-derived EVs using RT-qPCR and flow cytometry. n = 3-4 per each group. (E-I) The confirmation on the requirement of vesicular structures on the effects of NDTRs. (E) NTA analysis of lysed NDTRs. Left panel, the concentration of untouched neutrophil-derived EVs and lysed neutrophil-derived EVs. Right panels, Histogram depicting size distribution and concentration of untouched neutrophil-derived EVs and lysed neutrophil-derived EVs. Neutrophil-derived EVs were lysed with SDS. n = 4 per each group. (F) Confirmation of lysed neutrophil-derived EVs using flow cytometry. Neutrophil-derived EVs were stained with Anx_5, treated with either vehicle or SDS, and subjected to flow cytometric analysis. (G-I) The investigation on the effector functions of neutrophil-derived EVs (NDEVs) using annexin inhibition ($AnxA_5$ and $AnxA_1$), lysis (Lysed), filtration (Filtrated), and transwell (Transwell). The expressions of iNOS and Arg-1 in M0-differentiated THP-1 cells exposed to either NDTRs (G) or NDMVs (H). For comparison, M0-differentiated THP-1 cells were differentiated into M1 and M2 macrophages. (I) The effects of neutrophil-derived EVs on bacterial growth. NDEV + $AnxA_5$ denotes neutrophil-derived EVs treated with annexin A_5 antibody; NDEV + $AnxA_1$ denotes neutrophil-derived EVs treated with anti-annexin A_1 antibody; NDEV (lysed) denotes neutrophil-derived EVs lysed with SDS; NDEV (filtrated) denotes filtered supernatants from NDEVs. Data are shown as the mean ± SEM. *P < 0.05; **P < 0.01; ***P < 0.001.

A

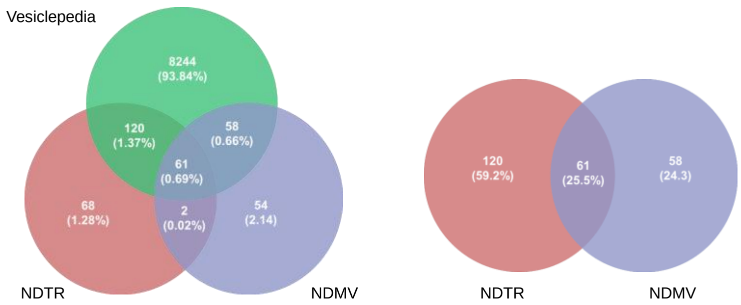

B

Category	NDMVs	Both	NDTRs
Cell/ tissue specific	ITAL	ITAM ITA2B ITB2 GNA12 GNAI2 GNAI3 GNAO	CD59 PTPRC SHH
Cytosolic	ANXA5 FLOT2	ANXA1 ANXA3 ANXA4 ANXA6 ACTA ACTB ACTC ACTG ACTH	HSP76
Secretory proteins	S10AB	CAP7 DEF1 DEF3 CATG ELNE LYSC NGAL PERE PERM PRTN3 TRFL	BPI
other intracellular compartments	CALX	CY24A CY24B ACTN1 ACTN	LAMB1 LAMC1

C

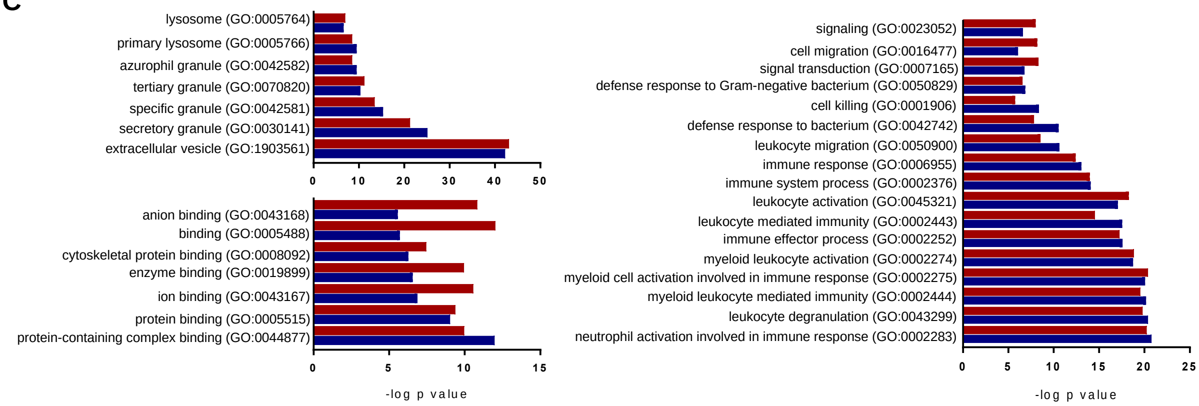

Figure S5. Related to Figure 5. Proteomic analysis of NDMVs and NDTRs. (A) Venn diagram showing difference in the proteins identified in NDMVs and NDTRs with respect to vesiclepedia (Left panel) and with respect to each other (Right panel) (B) Summary of proteins identified in NDMVs and NDTRs. (C) Functional enrichment analysis of identified proteins from NDTRs and NDMVs. Major GO biological processes (GOBP), GO molecular functions (GOMF) and GO cellular components (GOCC) identified using PANTHER overrepresentation, GO enrichment analysis (FDR < 0.001). n = 3 per each group.

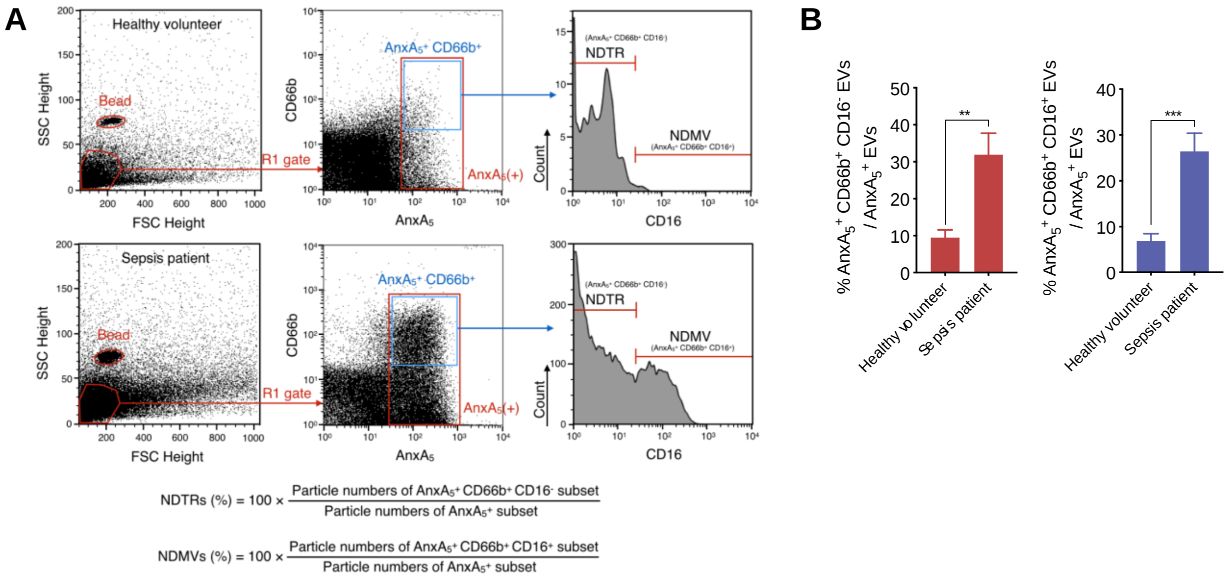

Figure S6. Related to Figure 6. The presence of neutrophil-derived EVs in plasma of sepsis patients. (A-B) EVs were collected from serum isolated from either healthy volunteer (n = 4) or septic patients (n = 12) using ExoQuick. EVs were stained with annexin A_5 antibody (FITC), anti-CD16 antibody (PE), and anti-CD66b antibody (PerCP). (A) Gating strategy for evaluation of neutrophil-derived EVs in human serum. EVs were gated as R1 based on 1 μm bead and $AnxA_5^+$ subset was considered as EVs. EVs were further gated according to CD66b expression and the expression level of CD16 was employed to discriminate NDTRs and NDMVs. The percentage of NDTRs and NDMVs was calculated as described. (B) The identification of neutrophil-derived EVs in plasma of sepsis patients. The percentages of neutrophil-derived EVs over total EVs was examined. Data are shown as the mean ± SEM. **P < 0.01; ***P < 0.001.
